# Neutrophils mediate *Salmonella* Typhimurium clearance through the GBP4 inflammasome-dependent production of prostaglandins

**DOI:** 10.1038/ncomms12077

**Published:** 2016-07-01

**Authors:** Sylwia D. Tyrkalska, Sergio Candel, Diego Angosto, Victoria Gómez-Abellán, Fátima Martín-Sánchez, Diana García-Moreno, Rubén Zapata-Pérez, Álvaro Sánchez-Ferrer, María P. Sepulcre, Pablo Pelegrín, Victoriano Mulero

**Affiliations:** 1 Facultad de Biología, Departamento de Biología Celular e Histología, Universidad de Murcia, IMIB-Arrixaca, 30100 Murcia, Spain; 2 Instituto de Investigaciones Marinas, CSIC, 36208 Vigo, Spain; 3 Unidad de inflamación y Cirugía Experimental, CIBERehd, Hospital Clínico Universitario Virgen de la Arrixaca, IMIB-Arrixaca, 30120 Murcia, Spain; 4 Facultad de Biología, Departamento de Bioquímica y Biología Molecular A, Universidad de Murcia, IMIB-Arrixaca, 30100 Murcia, Spain

## Abstract

Inflammasomes are cytosolic molecular platforms that alert the immune system about the presence of infection. Here we report that zebrafish guanylate-binding protein 4 (Gbp4), an IFNγ-inducible GTPase protein harbouring a C-terminal CARD domain, is required for the inflammasome-dependent clearance of *Salmonella* Typhimurium (ST) by neutrophils *in vivo*. Despite the presence of the CARD domain, Gbp4 requires the universal inflammasome adaptor Asc for mediating its antibacterial function. In addition, the GTPase activity of Gbp4 is indispensable for inflammasome activation and ST clearance. Mechanistically, neutrophils are recruited to the infection site through the inflammasome-independent production of the chemokine (CXC motif) ligand 8 and leukotriene B4, and then mediate bacterial clearance through the Gbp4 inflammasome-dependent biosynthesis of prostaglandin D2. Our results point to GBPs as key inflammasome adaptors required for prostaglandin biosynthesis and bacterial clearance by neutrophils and suggest that transient activation of the inflammasome may be used to treat bacterial infections.

## Abstract (web summary)

The role of guanylate-binding proteins (GBPs) in innate immunity is increasingly recognized. Here the authors show that GBP4 activates inflammasome in zebrafish neutrophils, and that this process is critical for the clearance of *Salmonella* infection via prostaglandin D2.

## Graphical abstract

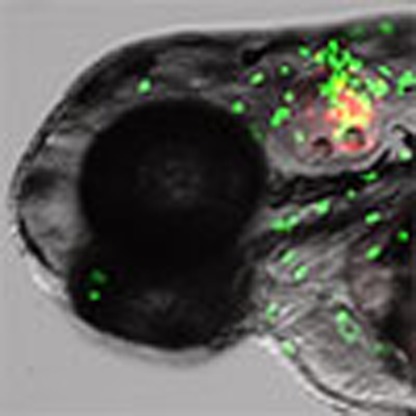

The nucleotide-binding domain leucine-rich repeats receptors (NLRs) constitute a family of cytosolic pattern recognition receptors, which are responsible for the caspase-1-mediated processing and activation of pro-inflammatory cytokines, such as interleukin-1β (IL-1β) and IL-18, and the induction of a special programme of cell death called pyroptosis, which is also dependent on caspase-1 (ref. 1). NLRs achieve these functions by oligomerizing into multiprotein signalling platforms, called inflammasomes, by their analogy with the apoptosome^1^,^2^. The NLR family, CARD domain containing 4 (NLRC4) detects bacterial flagellin and the basal body rod component of bacterial types III and IV secretion systems (T3SS and T4SS) of *Salmonella enterica* serovar Typhimurium (ST) and other flagellated bacteria^3^,^4^. When flagellin expression is inhibited, the NLR family pyrin domain (PYD) containing 3 (NLRP3) inflammasome also contributes to host defense during systemic ST infection^5^. Notably, NLRC4-mediated pyroptosis is required for the clearance of intracellular bacteria, while the processing of IL-1β seems to be dispensable^6^. More recently, the NLRC4 inflammasome has also been shown to generate a storm of eicosanoids through the activation of cytosolic phospholipase A_2_ (PLA2) in resident peritoneal macrophages^7^. However, the role of NLRC4 inflammasome-derived lipid mediators in pathogen clearance has not been examined.

The activation of most inflammasomes requires the adaptor molecule apoptosis-associated speck-like protein containing a CARD (ASC, also known as PYCARD), which binds to oligomerized NLRP proteins through homotypic PYD domain interaction leading to prion-like polymerizing structures that finally recruit pro-caspase-1 by its CARD domain, being necessary this coalition to convert pro-caspase-1 into its active form^8^,^9^. The NLRC4 inflammasome is a representative ASC-independent inflammasome, since NLRC4 contains a CARD that can directly recruit and activate caspase-1. However, ASC is required for some of the responses driven by NLRC4 (refs 10, 11). In addition, a recent study with confocal and superresolution microscopy has shown in macrophages infected with ST that ASC forms an outer ring-like structure that comprises NLRC4, NLRP3, caspase-1, caspase-8 and pro-IL-1β within the same macromolecular complex^12^.

Interferon γ (IFNγ)-inducible GTPases are highly evolutionary conserved proteins that operate cell-autonomously to defend vertebrate cells against a diverse group of invading pathogens^13^. They regulate vesicular trafficking and assembly of protein complexes to stimulate oxidative, autophagic and membranolytic-related antimicrobial activities within the cytosol, as well as on pathogen-containing vacuoles^14^. An elegant study using small interfering RNAs against the complete human and mouse GBP families in IFNγ/LPS/ATP treated macrophages has recently identified that guanylate-binding protein 5 (GBP5) is necessary for the specific activation of the NLRP3 inflammasome by live bacteria and their cell wall components, but not by crystalline agents or double-stranded DNA (ref. 15). Although this effect seems to be mediated by the direct promotion of the NLRP3-ASC inflammasome assembly by GBP5 (ref. 15), the mechanism orchestrating these interactions is largely unknown. Strikingly, GBP5 mutant mice show higher susceptibility to *Listeria monocytogenes*, phenocopying the pharmacological inhibition of caspase-1 in wild-type (WT) mice. In contrast, caspase-1 inhibition failed to affect the susceptibility of GBP5-deficient mice, suggesting that GBP5 promotes caspase-1-mediated protection against *L. monocytogenes* infection^15^. This has now been extended to *Francisella norvicida* where loss of GBP5 also impacts AIM2-dependent clearance of bacterial infection^16^.

Here we report in the zebrafish that, Gbp4, an IFNγ-inducible GTPase harbouring an N-terminal GTPase and C-terminal CARD domains is expressed in neutrophils and is required for the inflammasome-dependent clearance of ST *in vivo* via a different mechanism involving prostaglandins (PGs). Despite the presence of the CARD domain, Gbp4 unexpectedly requires the universal inflammasome adaptor Asc for mediating its antibacterial function. In addition, the GTPase activity of Gbp4 is also indispensable for inflammasome assembly, caspase-1 activation and resistance to ST, in contrast to mammalian GBP5 which is nonetheless able to rescue the higher bacterial susceptibility of Gbp4-deficient fish. Finally, we demonstrate that neutrophils are recruited to the infection site through the inflammasome-independent production of CXCL8 and LTB4 where they then mediate bacterial clearance through the Gbp4 inflammasome-dependent biosynthesis of PGs.

## Results

### Zebrafish Gbp4 is expressed in neutrophils

**Figure 1:**
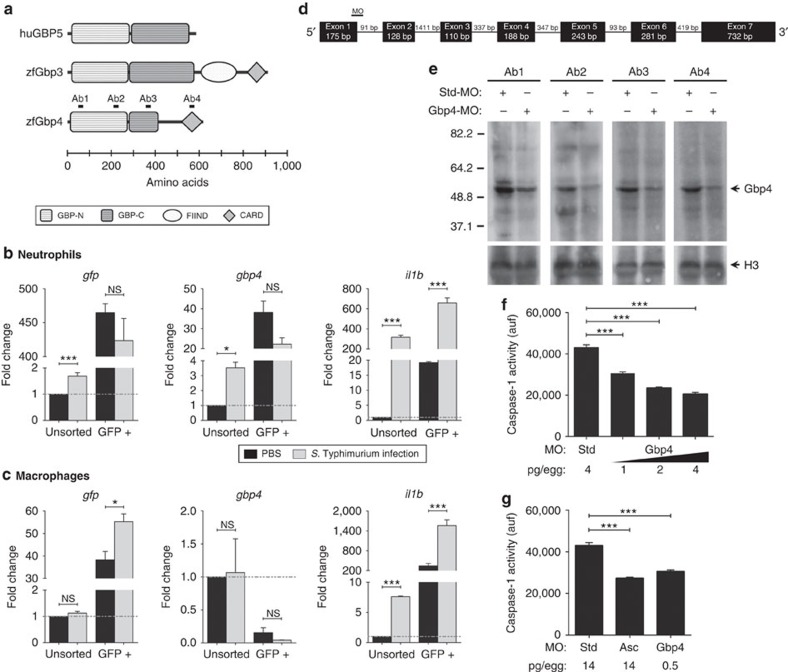
Zebrafish Gbp4 has functional GBP and CARD domains, and is highly expressed in neutrophils but not in macrophages. (**a**) Diagrams showing the domain organization of human GBP5 (NP_443174), and zebrafish Gbp3 (XP_009297061) and Gbp4 (NP_001076414). The guanylate-binding protein domain (GBP) is shown as open boxes (GBP, N-terminal, Pfam accession number PF02263) and grey boxes (GBP, C-terminal, Pfam accession number PF02841), the caspase recruitment domain (CARD, Pfam accession number PF00619) is shown as grey rhombus, and the function to find (FIIND, Pfam accession number PF13553) is shown as a grey circle. The position of each domain is indicated with respect to a ruler. The epitopes recognized by the different anti-Gbp4 antibodies used in this study are indicated. (**b**,**c**) *gfp, gbp4 and il1b* mRNA levels were measured by quantitative PCR with reverse transcription (RT-qPCR) in FACS-sorted neutrophils from *mpx:eGFP* (**b**) and macrophages from *mpeg1:eGFP* (**c**) 3 dpf larvae which were previously infected with ST or not at 2 dpf. Data were normalized with unsorted, uninfected cells. (**d**) Diagram showing the exons/introns organization of zebrafish *gbp4* gene, indicating the position where the Gbp4 MO binds to the pre-mRNA. (**e**–**g**) Zebrafish one-cell embryos were injected with the indicated concentrations of standard control (std), Asc or Gbp4 MOs. Total proteins were isolated from dechorionated and deyolked larvae at 3 dpf, and the efficiency of the Gbp4 MO was validated using western blot with four different antibodies. (**f**,**g**). Caspase-1 activity levels were measured at 3 dpf in larvae infected with ST at 2 dpf. Each bar represents the mean±SEM of triplicate samples from pooled larvae. The sample size for each treatment is 500 in **b** and **c**, 50 in **e**, 30 in **f** and **g**. NS, not significant; *P<0.05; ***P<0.001 according to analysis of variance (ANOVA) and Tukey multiple range test.

**Figure 2:**
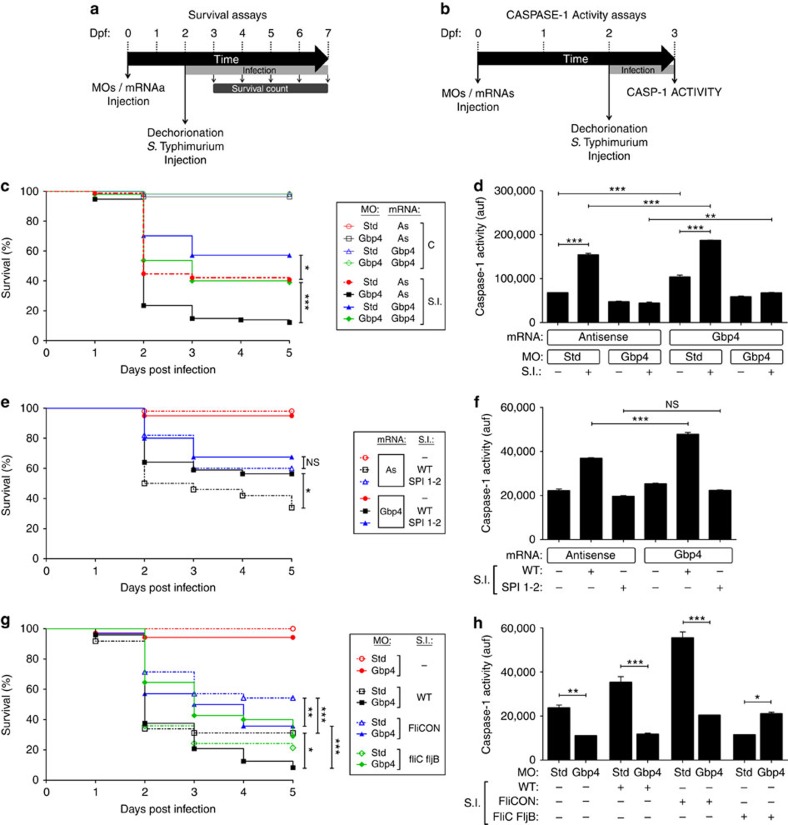
Zebrafish Gbp4 is required for the inflammasome-dependent resistance to *S*. Typhimurium. (**a**) Scheme showing the experimental procedure used for the survival assays. Zebrafish one-cell embryos were injected with MOs and/or mRNAs, dechorionated and infected at 2 dpf via the yolk sac with different strains of ST at a multiplicity of infection (MOI) of 10, and the number of surviving larvae counted daily during the next 5 days. At least 3 independent experiments were performed with a total number of >300 specimens/treatment (actual number indicated in each legend). (**b**) Scheme showing the experimental procedure used for the caspase-1 activity assays. Zebrafish one-cell embryos were injected with MOs and/or mRNAs, dechorionated and infected at 2 dpf via the yolk sac with different strains of ST with a MOI of 50, and collected 24 hpi and pooled (25–35 larvae) to measure caspase-1 activity. At least three independent experiments were performed and one of them is shown. (**c**,**d**) Zebrafish one-cell embryos were injected with standard control (std) or Gbp4 MOs in combination with antisense (As) or Gbp4 mRNAs, infected at 2 dpf with WT ST (**d**,**e**), WT and the DM SPI 1–2 (**e**,**f**), or (WT), FliCON and FliC FljB (**g**,**h**), and the survival (**c**,**e**,**g**) and caspase-1 activity (**d**,**f**,**h**) were determined as described in **a** and **b**, respectively. The sample size for each treatment is 325 in **c**, **e** and **g**, 30 in **d**, **f**, and **h**. NS, not significant; SI, ST infection; *P<0.05; **P<0.01; ***P<0.001 according to log-rank test (**c**,**e** and **g**) or ANOVA followed by Tukey multiple range test (**d**,**f** and **h**).

The zebrafish genome contained two annotated genes that encode two GBP proteins, termed Gbp3 and Gbp4, with N-terminal GBP and C-terminal CARD domains (Fig. 1a), a configuration first highlighted by Shenoy *et al.*^15^ in their discovery of GBP-mediated inflammasome regulation and absent in mammals. Gbp3 showed 23 and 35% amino acid homology and identity with human GBP5, while Gbp4 had 36 and 53% amino acid homology and identity with human GBP5. We decided to examine the gene expression profile and function of zebrafish Gbp4. Neutrophils and macrophages were FACS-sorted from *mpx:eGFP* (ref. 17) and *mpeg1:eGFP* (ref. 18) transgenic lines on infection with ST, respectively, and it was found that Gbp4 transcripts were highly enriched in neutrophils, while they were hardly detected in macrophages (Fig. 1b,c). In addition, infection with ST had negligible effects in the mRNA levels of Gbp4 in both neutrophils (Fig. 1b) and macrophages (Fig. 1c), while both cells showed increased mRNA levels of *il1b* on infection (Fig. 1b,c). We then used a morpholino (MO)-mediated gene knockdown strategy to target the exon 1/intron 1 boundary and altered the splicing of Gbp4 mRNA (Fig. 1d). The efficiency of the MO against Gbp4 was checked by western blot using four different monoclonal antibodies (mAbs) targeting both domains of the protein (Fig. 1a and Supplementary Fig. 1) and it was confirmed by a strong reduction of Gbp4 protein in Gbp4 morphants compared with control morphants (Fig. 1e). We next evaluated caspase-1 activity using a fluorometric substrate, Z-YVAD-AFC, which has been previously shown to be processed by fish native and recombinant caspase-1 (refs 19, 20). The results showed a dose-dependent inhibition of basal caspase-1 activity in larvae injected with increased concentrations of the Gbp4 MO compared with controls (Fig. 1f), the inhibition reaching similar levels to the one achieved with a MO targeting the exon 2-intron 2 boundary of the mRNA of the inflammasome adaptor protein Asc (Fig. 1g). No developmental defects or mortality were observed in Gbp4 (Fig. 2) morphant animals injected with 0.5–1 pg per egg MOs, so this dose was used in the following experiments.

### Gbp4 is required for the resistance to ST

Due to its clinical importance, ST has been widely used as a model organism for the study of host–pathogen interactions and, in particular, to the role of the inflammasome in the clearance of intracellular bacteria. The virulence of ST is linked to their two Salmonella pathogenicity islands, termed SPI-1 and SPI-2, which contain a large number of genes encoding a T3SS. The function of SPI-1 appears to be required for the initial steps of systemic infections^21^ and, although the molecular functions of SPI-2 has not been characterized in detail, SPI-2 mutants are severely attenuated in virulence in a mouse model of systemic infection and fail to proliferate in infected host organs, like the liver and spleen^22^. Infection of zebrafish larvae with WT ST resulted in a high mortality using the yolk sac as the route of infection (Supplementary Fig. 2A). In contrast, a syngenic double mutant (DM) for SPI-1 and SPI-2 showed reduced virulence, while single SPI-1 or SPI-2 mutants had an intermediate virulence (Supplementary Fig. 2A). Notably, WT ST induced the activation of caspase-1, while single or double SPI mutants failed to do so (Supplementary Fig. 2B). These results demonstrate the usefulness of this model to ascertain the role of SPI in bacterial virulence in a whole-vertebrate organism in the absence of adaptive immunity. So, we next infected Gbp4-deficient fish with WT ST and found an increased susceptibility to the infection compared with their control siblings (Fig. 2a,c) and impaired caspase-1 activity in response to the infection (Fig. 2b,d). In addition, bacterial susceptibility, and to some extent caspase-1 activity, were reversed by injection of non-targetable Gbp4 mRNA. More interestingly, forced expression of Gbp4 RNA alone increased resistance to the infection (Fig. 2c), caspase-1 activity (Fig. 2d) and ST clearance (Supplementary Fig. 3), confirming the specificity of the MO and revealing the crucial role of Gbp4 in the resistance to this infection. In contrast, Gbp4 overexpression did not affect fish susceptibility to the SPI-1/SPI-2 ST mutant (Fig. 2e,f). Notably, detection of ST in zebrafish did not depend on flagellin as WT and a syngenic mutant strain lacking flagellin (FliC/FljB)^23^ showed a similar survival in WT fish (Fig. 2g,h). When FliC was overexpressed in ST (FliCON)^6^ there was an increase of larvae survival that was GBP4-dependent (Fig. 2g,h), suggesting that GBP4 functions downstream of FliC detection.

**Figure 3:**
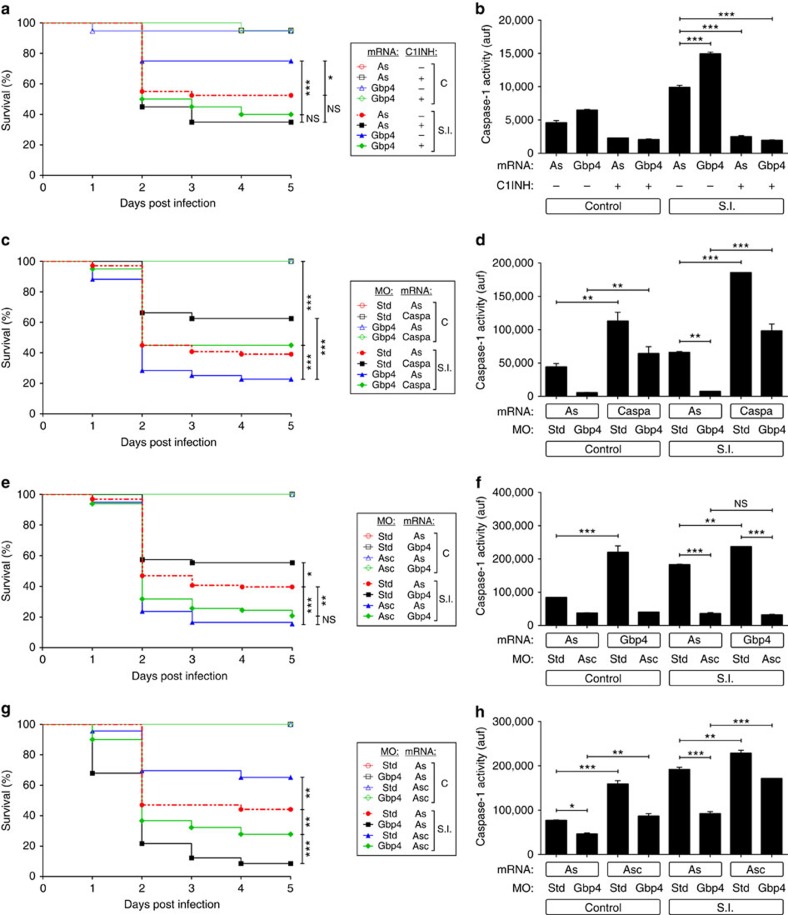
The Gbp4-mediated resistance to *S.* Typhimurium is Caspa and Asc-dependent. (**a-b**) Zebrafish one-cell embryos were injected with antisense (As) or Gbp4 mRNAs and then treated by immersion with vehicle alone (DMSO) or 100 μM of a specific inhibitor of caspase-1 (Ac-YVAD-CMK,C1INH). (**c**,**d**) Zebrafish one-cell embryos were injected with standard control (std) or Gbp4 MOs in combination with antisense (As) or Caspa mRNAs (**c**,**d**). At 2 dpf, embryos were infected and survival (**a**,**c**) and caspase-1 activity (**b**,**d**) determined as described in Fig. 2a,b, respectively. (**e**–**h**) Zebrafish one-cell embryos were injected with standard control (std), Asc (**e**,**f**) or Gbp4 MOs (**g**,**h**) in combination with antisense (As), Gbp4 (**e**,**f**) or Asc (**g**,**h**) mRNAs, infected at 2 dpf and survival (**e**,**g**) and caspase-1 activity (**f**,**h**) determined as described in Fig. 2a,b, respectively. The sample size for each treatment is 300 in **a**, **c**, **e** and **g**, 30 in **b**, **d**, **f** and **h**. NS, not significant; SI, ST infection; *P<0.05; **P<0.01; ***P<0.001 according to log-rank test (**a**,**c**,**e** and **g**) or ANOVA followed by Tukey multiple range test (**b**,**d**,**f** and **h**).

Next, we used the pan-caspase inhibitor Q-VD-OPh (PINH; Supplementary Fig. 4A,B) as well as the specific caspase-1 inhibitor Ac-YVAD chloromethylketone (Ac-YVAD-CMK, C1INH; Fig. 3a,b), which was shown to inhibit the activity of recombinant fish caspase-1 (refs 19, 20), and it was found that bath treatment with either inhibitor increased the susceptibility of zebrafish larvae to ST infection and, more importantly, fully abrogated the Gbp4-mediated increased infection resistance (Supplementary Figs 3A and 4A). As expected, both inhibitors strongly decreased caspase-1 activity *in vivo* (Supplementary Figs 3B and 4B).

Although a homologue of mammalian caspase-1 has not been identified in the zebrafish genome to date, it has been reported the existence of caspase a (Caspa, also known as Caspy) and caspase b (Caspb, also known as Caspy2), both of them harbouring an N-terminal PYD^24^. While Caspa preferentially cleaves AcYVAD-AMC, a caspase-1 substrate, and interacts and co-localizes to the speck with zebrafish Asc when ectopically expressed in human embryonic kidney 293 T (HEK293T) cells, Caspb was more active on AcWEHD-AFC, a preferred substrate of caspase-5, and it did not interact with Asc (ref. 24). We, therefore, analysed whether Caspa and Caspb were able to rescue the high susceptibility to ST of Gbp4-deficient larvae. Caspa was able to fully rescue the susceptibility (Fig. 3c) and partially caspase-1 activity (Fig. 3d) of Gbp4-deficient larvae, while it failed to do so in Asc-deficient larvae (Supplementary Figs 4C,D). However, forced expression of Caspb resulted in developmental defects in most injected fish and the ones normally developed showed unaltered susceptibility to ST (Supplementary Fig. 4E) and slightly higher caspase-1 activity than control fish (Supplementary Fig. 4F). In addition, Caspb failed to rescue the caspase-1 activity of Gbp4-deficient larvae (Supplementary Fig. 4F). Collectively, these results confirm that Caspa is the functional homologue of mammalian caspase-1 and acts downstream of Asc *in vivo*.

The presence of a CARD in zebrafish Gbp4 suggests that it may directly recruit and activate caspase-1 without the need of the adaptor Asc. So, we knocked down Asc using the exon 2/intron 2 splice-blocking MO. PCR with reverse transcription analysis showed that the MO was able to alter the splicing of *asc* transcripts up to 6 days post-fertilization (dpf; Supplementary Fig. 5A). Genetic depletion of Asc in zebrafish had no apparent effects on larval development but drastically increased the susceptibility of larvae to ST and, strikingly, completely abrogated the Gbp4-mediated increased infection resistance (Fig. 3e). In addition, we observed that Asc deficiency significantly reduced endogenous and ST- and Gbp4-induced caspase-1 activity (Fig. 3f). Similar results were obtained with a translation-blocking MO (Supplementary Fig. 5B,C). Conversely, forced expression of Asc strongly increased larval resistance to the infection and caspase-1 activity, these effects being largely independent of Gbp4 (Fig. 3g,h). The relevance of Asc in the clearance of ST was further confirmed by the ability of a mutant zebrafish Asc, harbouring a C-terminal green fluorescent protein (GFP) instead of its CARD, to behave as dominant negative (DN) by increasing larval susceptibility to ST (Supplementary Fig. 5D). Collectively, these results show that Gbp4 requires Asc to induce inflammasome activation and caspase-1-dependent resistance to ST.

### The GTPase activity of Gbp4 is required for resistance to ST

**Figure 4:**
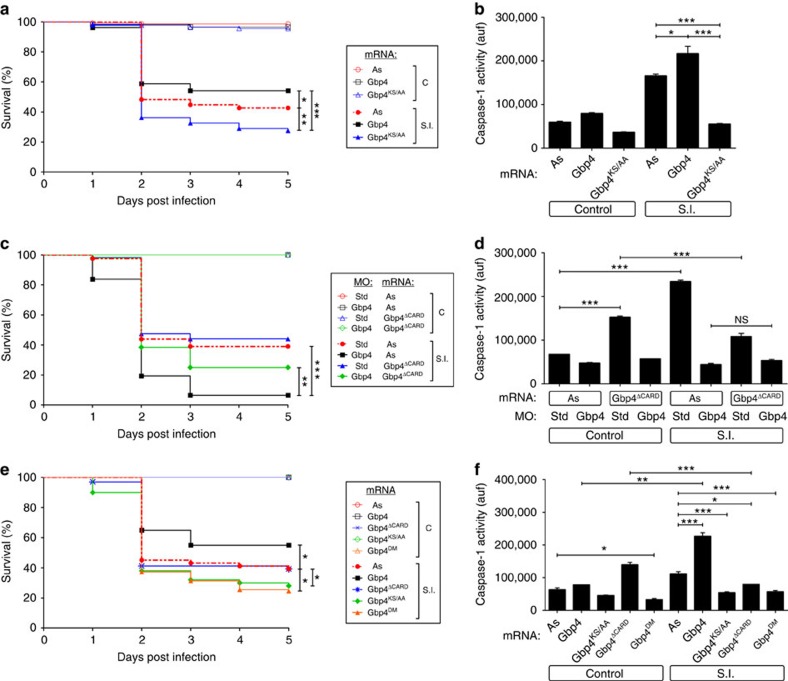
Zebrafish Gbp4 requires its GTPase activity for the inflammasome-dependent resistance to *S*. Typhimurium. Zebrafish one-cell embryos were injected with antisense (As), Gbp4 (**a**,**b**,**e** and **f**), Gbp4^KS/AA^ (**a**,**b**,**e** and **f**), Gbp4^ΔCARD^ (C-F) or Gbp4^DM^ (**e**,**f**) mRNAs in combination with standard control (std) or Gbp4 MOs (C, D), infected at 2 dpf, and survival (**a**) and caspase-1 activity (**b**) determined as described in Fig. 2a,b, respectively. (**c**,**d**) Zebrafish one-cell embryos were injected with antisense (As) or Gbp4^ΔCARD^ mRNAs, infected at 2 dpf, and survival (**c**) and caspase-1 activity (**d**) determined as described in Fig. 2a,b, respectively. (**e**,**f**) Zebrafish one-cell embryos were injected with antisense (As), Gbp4, mRNAs, infected at 2 dpf, and survival (**e**) and caspase-1 activity (**f**) determined as described in Fig. 2a,b, respectively. The sample size for each treatment is 340 in **a**, **c** and **e**, 30 in **b**, **d** and **f**. NS, not significant; SI, ST infection; *P<0.05; **P<0.01; ***P<0.001 according to log-rank test (**a**,**c** and **e**) or ANOVA followed by Tukey multiple range test (**b**,**d** and **f**).

**Figure 5:**
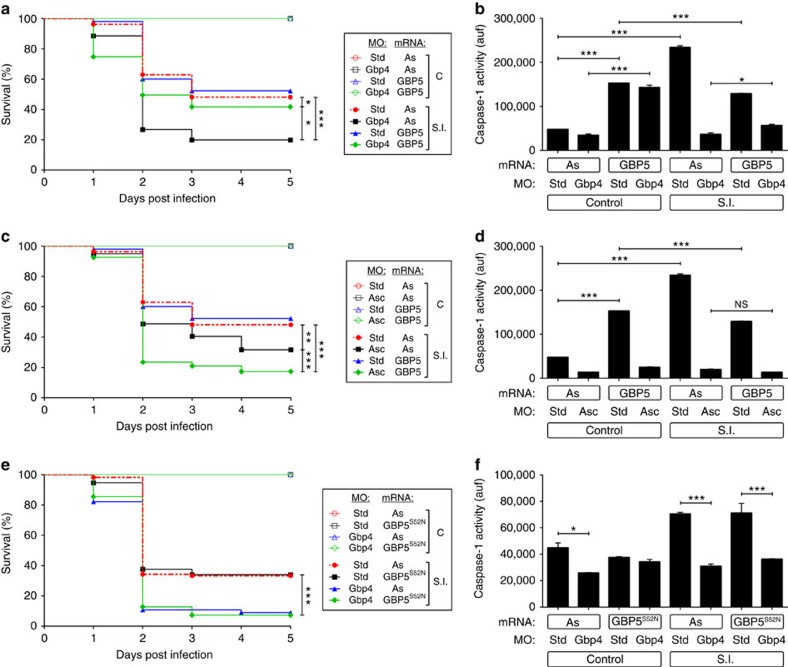
Mouse GBP5 rescues the high *S*. Typhimurium susceptibility of Gbp4-deficient zebrafish larvae in Asc-dependent manner. Zebrafish one-cell embryos were injected with standard control (std), Gbp4 (**a**,**b**,**e** and **f**) or Asc (**c**,**d**) MOs in combination with antisense (As), GBP5 (**a**–**d**) or GBP5^S52N^ (**e**,**f**) mRNAs, infected at 2 dpf, and survival (**a**) and caspase-1 activity (**b**) determined as described in Fig. 2a,b, respectively. (**c**,**d**). The sample size for each treatment is 340 in **a**, **c** and **e**, 30 in **b**, **d** and **f**. NS, not significant; SI, ST infection; *P<0.05; **P<0.01; ***P<0.001 according to log-rank test (**a**,**c** and **e**) or ANOVA followed by Tukey multiple range test (**b**,**d** and **f**).

Although mammalian GBP1 displays GTPase-dependent tetramerization, both recombinant WT GBP5 and the GTPase-deficient mutant (GBP5KS→AA) formed tetramers and are equally able to promote Asc multimerization^15^. However, ASC assembly was abolished when two tetramerization GBP5 mutants were used, suggesting that tetrameric GBP5 promotes ASC oligomerization^15^. We generated a similar GTPase-deficient mutant of zebrafish Gbp4 (Gbp4KS→AA) and unexpectedly found that it was not only unable to increase the resistance of zebrafish to ST infection but rather behaved as a DN that increased susceptibility to the infection (Fig. 4a) and concomitantly inhibited caspase-1 activity (Fig. 4b) and impaired ST clearance (Supplementary Fig. 3). We then tested the impact of a Gbp4 mutant devoid of its CARD (Gbp4ΔCARD) and observed that although it was able to partially rescue the higher susceptibility of Gbp4-deficient larvae, it failed to increase their infection resistance when expressed alone (Fig. 4c). In addition, it negatively affected the activation of caspase-1 in infected larvae (Fig. 4d). On the other hand, a DM form devoid of both GTPase activity and the CARD behaved as the GTPase-deficient mutant (Fig. 4e,f). Strikingly, mouse GBP5 was able to rescue both higher ST susceptibility (Fig. 5a) and caspase-1 defects (Fig. 5b) of Gbp4-deficient larvae. However, it was unable to increase the larval infection resistance when expressed alone, even though it increased caspase-1 activity in non-infected animals (Fig. 5a,b). Notably, all these effects required zebrafish Asc (Fig. 5c,d), as reported in mammals^15^. Notably, the GTPase-deficient mutant GBP5(S52N) failed to rescue the hypersusceptibility to ST (Fig. 5e) and to restore caspase-1 activity levels (Fig. 5f) in Gbp4-deficient larvae. In addition, it did not show a DN effect, in contrast to zebrafish Gbp4KS→AA (Fig. 5e,f). Collectively, these results indicate that mammalian GBP5 behaved like zebrafish Gbp4ΔCARD and suggest that the CARD of zebrafish Gbp4 conferred its ability to promote caspase-1-mediated resistance to intracellular bacteria. Supporting this hypothesis, expression of increasing amounts of WT Gbp4 promoted a dose-dependent activation of caspase-1 in both control and infected larvae (Supplementary Fig. 6A), while as expected, Gbp4KS→AA and Gbp4DM impaired the activation of caspase-1 in both conditions (Supplementary Fig. 6B,D), confirming their DN effect. However, Gbp4ΔCARD promoted a dose-dependent caspase-1 activation in non-infected larvae, while inhibited its activation on infection (Supplementary Fig. 6C).

**Figure 6:**
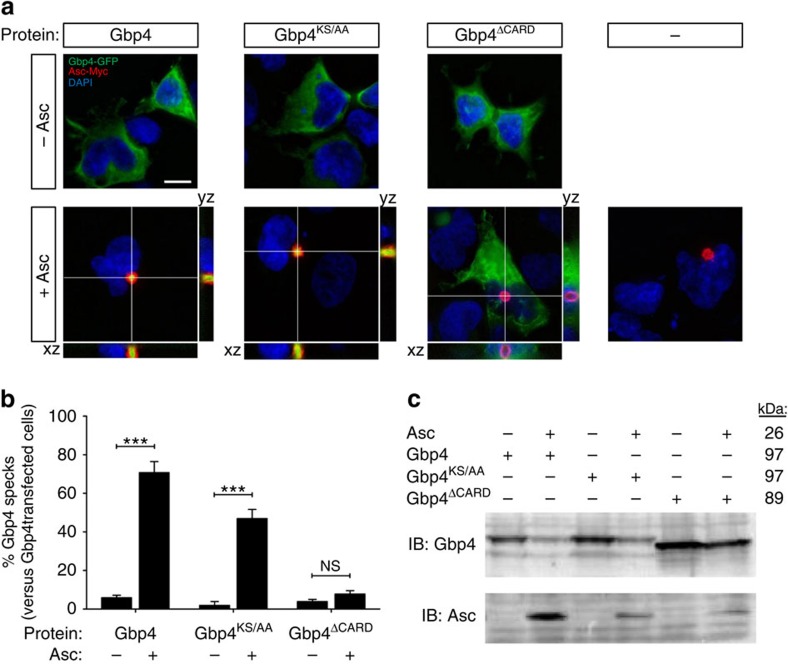
Gbp4 and Gbp4^KS/AA^ localize to the speck in the presence of Asc. HEK293T cells were transfected with zebrafish Gbp4-GFP, Gbp4^KS/AA^-GFP or Gbp4^▵CARD^-GFP in the presence or absence of zebrafish Asc-Myc, fixed at 48 h post-transfection and labelled in red with specific antibodies to the Myc epitope. (**a**) Representative frontal (xy) and lateral (xz and yz) views of maximum-intensity projection images of HEK293T cells stained with anti-Myc antibodies (Asc, Red). Gbp4, Gbp4^KS/AA^ and Gbp4^▵CARD^ are visualized in green thanks to GFP, while nuclei are labelled with DAPI (blue). (**b**) Quantitation of the percentage of Gbp4 specks in relation to the total number of Gbp4 transfected cells. (**c**) Cells were lysed and anti-GFP and anti-c-Myc antibodies were used to validate the transfection assays by detecting Gbp4 and Asc, respectively, by western blot. The mass weights for all the proteins are indicated. Scale bars, 10 μm. The sample size for each treatment is Gbp4: 1300 cells; Gbp4+Asc: 1270 cells; Gbp4^KS/AA^: 369 cells; Gbp4^KS/AA^ +Asc: 768 cells; Gbp4^▵CARD^: 773 cells; Gbp4^▵CARD^+Asc: 961cells. NS, not significant; ***P<0.001 according to ANOVA followed by Tukey multiple range test (B).

The above results suggest that not only WT Gbp4, but also the GTPase-deficient mutant, were able to interact with Asc and then trigger or block, respectively, inflammasome activation, caspase-1 activation and ST clearance. We tested this idea by reconstituting Gbp4-Asc complexes in HEK293T cells, which lack each of these components. Both WT and GTPase-deficient mutant Gbp4 fused to GFP were found to localize to the inner core of the Asc speck, while they appeared diffusely distributed in the cytosol in the absence of Asc (Fig. 6a–c). In addition, the CARD-deficient Gbp4 mutant was found distributed through the cytosol independently of Asc and unable to localize to the Asc speck (Fig. 6a,b). This result may be explained by the intrinsic six-helix bundle fold of CARD domains (Supplementary Fig. 7A) with prominent charged surface patches in both Gbp4 (Supplementary Fig. 7B) and Asc (Supplementary Fig. 7C), whose electrostatic attractions mediate their association in combination with hydrogen bonds, van der Waals forces and salt bridges, as it has been described for pro-caspase-1:huNLRP1 (ref. 25) and pro-caspase-9:Apaf-1 (ref. 26) interaction.

### Neutrophils mediate the Gbp4-dependent resistance to ST

**Figure 7:**
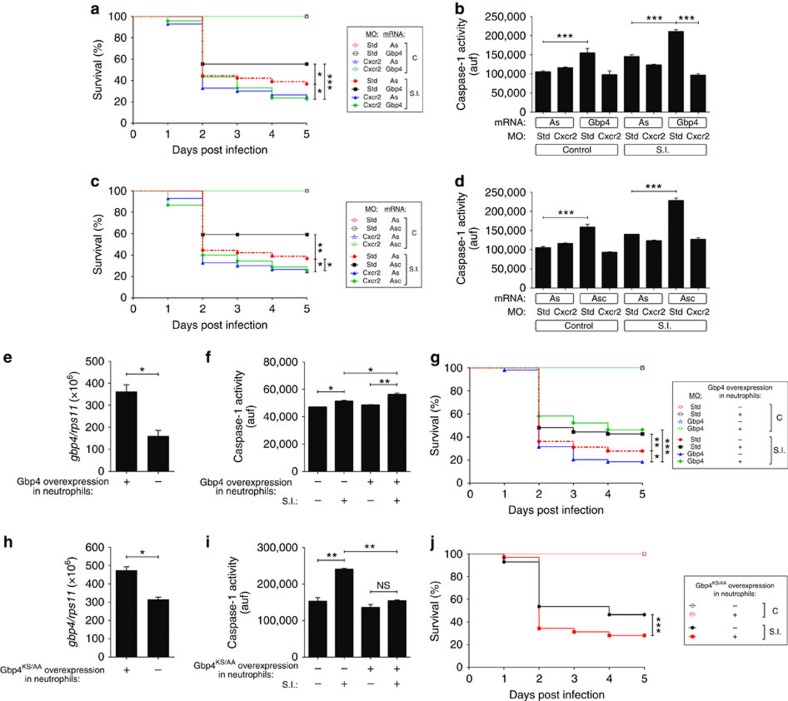
Neutrophils mediate the Gbp4-dependent resistance to *S*. Typhimurium. (**a**–**d**) Zebrafish one-cell embryos were injected with standard control (std) or Cxcr2 MOs in combination with antisense (As), Gbp4 (**a**,**b**) or Asc (**c**,**d**) mRNAs, infected at 2 dpf, and survival (**a**,**b**) and caspase-1 activity (**b**,**d**) determined as described in Fig. 2a,b, respectively. (**e**–**g**) Zebrafish WT (−) and their *mpx:Gal4.VP16; uas:gbp4* siblings (+) embryos were injected at the one-cell stage with standard control (std) or Gbp4 MOs, infected at 2 dpf with WT ST, and the mRNA levels of *gbp4* (**e**) and caspase-1 activity (**f**) were determined in whole larvae at 3 dpf. (**g**) The survival was also analysed as indicated in Fig. 2a. (**h–j**) Zebrafish WT (−) and their *mpx:Gal4.VP16; uas:gbp4*^*KS/AA*^ siblings (+) embryos were infected at 2 dpf with WT ST, and the mRNA levels of *gbp4*^*KS/AA*^ (**h**), caspase-1 activity (**i**) and the survival (**j**) were determined as indicated in Fig. 2a,b. The sample size for each treatment is 340 in **a**, **c**, **g** and **j**, 30 in **b**, **d**, **f** and **i**, 35 in **e** and **h**. auf, arbitrary units of fluorescence; NS, not significant; SI, ST infection; *P<0.05; **P<0.01; ***P<0.001 according to log-rank test (**a**,**c**,**g** and **j**), ANOVA followed by Tukey multiple range test (**b**,**d**,**f** and **i**) or Student's *t*-test (**e** and **h**).

As Gbp4 is highly expressed in neutrophils and these cells are essential for ST clearance in zebrafish^27^, we next examined the impact of Gbp4 in neutrophil development and functions. Gbp4-deficient larvae showed reduced number of neutrophils at 3 dpf, the effect being rescued by Gbp4 RNA (Supplementary Fig. 8A,B). Forced expression of granulocyte colony-stimulating factor a (Gcsfa, also known as Csf3a), drastically increased the resistance of WT larvae to ST infection, whereas it failed to rescue the high susceptibility to ST of their Gbp4-deficient siblings (Supplementary Fig. 8C). As expected, Gcsfa increased the number of neutrophils in both WT and Gbp4-deficient larvae (Supplementary Fig. 8D,E), demonstrating that the benefit of Gbp4 is independent of effects on neutrophil population. So, we blocked neutrophil recruitment to infection foci by using a specific MO to Cxcr2, which is responsible for IL-8-dependent neutrophil recruitment to ST in zebrafish^27^. The results showed that Cxcr2 deficiency resulted in higher susceptibility to the infection and, interestingly, it fully overcame both Gbp4- (Fig. 7a) and Asc- (Fig. 7c) induced infection resistance and caspase-1 activity (Fig. 7b,d). The relevance of neutrophils in mediating the Gbp4-dependent resistance to ST was further confirmed by the full rescue of the higher susceptibility phenotype of Gbp4-deficient larvae to ST in a transgenic line expressing WT Gbp4 specifically in neutrophils (Fig. 7e–g) and, conversely, by the higher susceptibility to ST and impaired caspase-1 activity of a transgenic line expressing DN Gbp4KS→AA in neutrophils (Fig. 7h–j). Collectively, these results indicate a pivotal role of Gbp4 in neutrophil homeostasis and functions in zebrafish and support the crucial role of these cells in the clearance of intracellular bacteria, as occurs in mammals^6^.

### Gbp4 does not regulate IL-1β processing and pyroptosis

The involvement of caspase-1 in the processing of IL-1β in fish is controversial^19^,^28^,^29^ and, in fact, all fish IL-1β sequenced to date lack a conserved caspase-1 processing site^30^. In addition, while ST fails to promote IL-1β processing in the teleost fish gilthead seabream^28^, recombinant Caspa and Caspb can process zebrafish IL-1β *in vitro*^31^ and pharmacological inhibition of caspase-1 attenuated neutrophil and macrophage migration to wound *in vivo*^32^. Therefore, we investigated whether ST infection triggered IL-1β processing in zebrafish. Unfortunately, processed IL-1β was not detected by western blot in whole-larval extracts using several mAbs, suggesting a rapid elimination of the mature cytokine *in vivo* and/or its restricted production at the infection foci. Therefore, we used a recently developed luciferase-based inflammasome and protease activity reporter assay (iGLuc), where inactive pro-IL-1β-*Gaussia* luciferase fusion needs to be processed by caspase-1 to render GLuc enzyme active^33^. Infection of larvae expressing a RNA encoding a fusion between zebrafish pro-IL-1β and GLuc (zfiGLuc) resulted in increased luciferase activity independently of Asc (assayed in Asc-deficient fish) and caspase-1 activity (assayed in the presence of caspase-1 or pan-caspase inhibitors; Supplementary Fig. 9A). As expected, the luciferase activity of native GLuc was unaffected by Asc deficiency or the presence of caspase inhibitors (Supplementary Fig. 9A), confirming the specificity of the assay in whole-zebrafish larvae. Similarly, Gbp4 levels did not affect the luciferase activity in fish expressing zfiGLuc (Supplementary Fig. 9B,C). Furthermore, genetic ablation of IL-1β using a specific MO (ref. 34) did not affect the larval resistance to ST or caspase-1 activity (Supplementary Fig. 9D,E), further suggesting that the Gbp4/Asc/Caspa-mediated resistance to ST is independent of IL-1β.

We then analysed whether ST infection resulted in pyroptotic cell death, as we have shown that infection of macrophages from the teleost fish gilthead seabream with WT ST, but not with its derivative isogenic SPI-1 mutant strain, triggers pyroptosis^28^. However, we did not find significant cell death of neutrophils in a localized ear infection model, assayed by YO-PRO uptake (Supplementary Fig. 9F).

### The Gbp4 inflammasome regulates prostaglandin biosynthesis

It has been shown that the activation of the mouse NLRC4 inflammasome results in the generation of a pathological storm of eicosanoids through the activation of cytosolic PLA2 (ref. 7), which is required for arachidonic acid (AA) release from the plasma membranes for eicosanoid biosynthesis (Supplementary Fig. 10). Gene expression analysis of infected neutrophils showed increased transcript levels of genes encoding cPla2 (Supplementary Fig. 11A), PG-endoperoxidase synthase 2 (Ptgs2, also known as cyclooxygenase 2), which is the inducible form of the enzyme involved in PGs biosynthesis, and Il1b (Supplementary Figs 10 and 11B–D). However, the transcript levels of the genes encoding arachidonate 5-lipoxygenase (Alox5), which is involved in leukotriene (LT) biosynthesis (Supplementary Fig. 10), and constitutive Ptgs1 both declined in neutrophils on ST infection (Supplementary Fig. 11E–G). Furthermore, *ptgs2a* mRNA levels, but not *ptgs1*, were higher at 1 hpi in larvae forced to express WT Gbp4 (Supplementary Fig. 11H,I). More importantly, infection resulted in increased Pla2 activity in whole larvae, being this activation impaired by pharmacological inhibition of caspase-1 (Supplementary Fig. 11J) and, conversely, further increased by forced expression of Gbp4 and Caspa but not Asc (Supplementary Fig. 11K).

**Figure 8:**
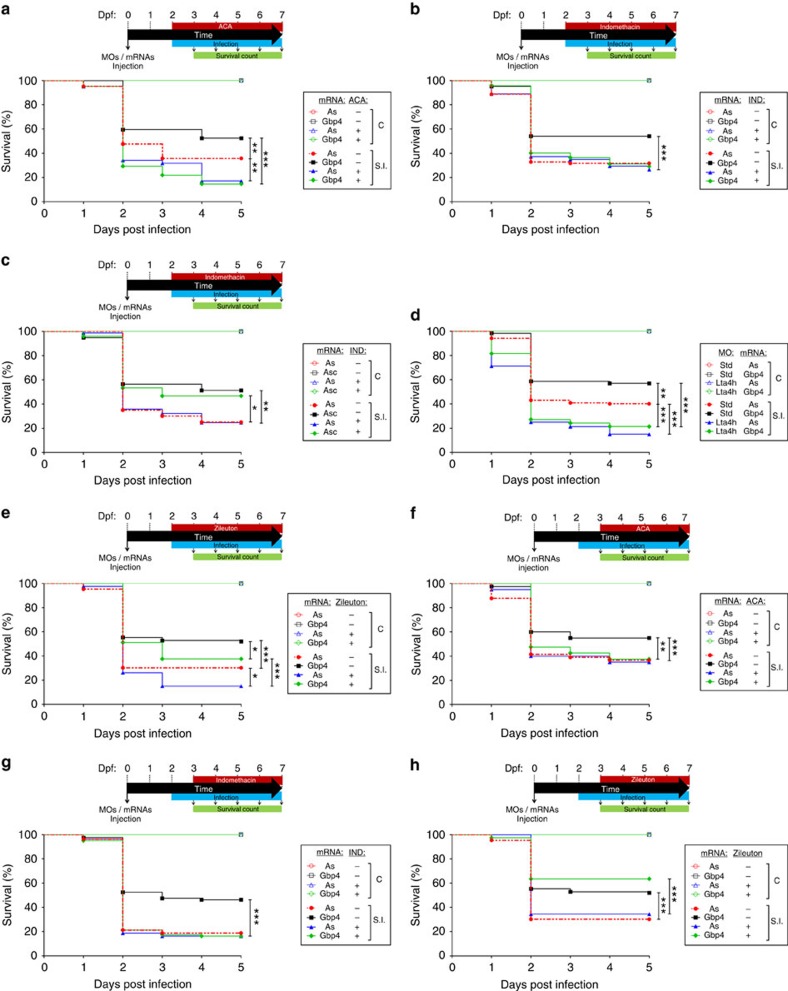
Two waves of different eicosanoids are required for the clearance of S. Typhimurium in vivo. (**a**–**h**) Zebrafish one-cell embryos were injected with antisense (As), Gbp4 (**a**,**b** and **d**–**h**) or Asc (**c**) mRNAs alone or combined with standard control (std) or Lta4h MOs (**d**). Larvae were then treated by immersion with pharmacological inhibitors of Pla2 (N-(p-Amylcinnamoyl) anthranilic acid, ACA, 0.5 μM; **a**,**f**), Ptgs1/2 (Indomethacin, IND, 10 μM) (**b**,**c** and **g**), Alox5 (zileuton, 20μM) (**e**,**h**) or vehicle alone (DMSO) at 2 dpf (1 h before infection) (**a**–**c**,**e**) or 3 dpf (24 hpi) (**f**–**h**), infected at 2 dpf and survival determined as described in Fig. 2a. The sample size for each treatment is 300 in **a**–**h**. SI, ST infection. *P<0.05; **P<0.01; ***P<0.001 according to log-rank test (**a**–**h**).

These results led us to analyse whether Gbp4/inflammasome/caspase-1-mediated resistance to ST involved the production of eicosanoids by manipulating eicosanoid biosynthesis 1 h before infection. Pharmacological inhibition of cPla2 impaired ST clearance in both WT and Gbp4 overexpressing larvae (Supplementary Figs 8A and 12A), while exogenous addition of AA increased resistance of both WT and Gbp4-deficient larvae (Supplementary Fig. 13A). However, pharmacological inhibition of PG production, that is, inhibition of Ptgs1 and 2 (Fig. 8b) or specifically Ptgs2 (Supplementary Fig. 12B), fully reversed the higher infection resistance of larvae forced to express Gbp4 but, unexpectedly, the resistance of larvae forced to express Asc was unaffected (Fig. 8c). In addition, inhibition of PG production did not affect the susceptibility of WT larvae (Fig. 8b,c and Supplementary Fig. 12B). The differential effects of cPla2 and Ptgs inhibitors suggest the requirement of LT in the clearance of ST but independently of inflammasome activation. As LTB4 has been reported to be one of the main modulators of neutrophil recruitment^35^,^36^, we also examined the impact of inhibiting its production by knocking down LTA4 hydrolase (Lta4h)^37^ or by inhibiting Alox5 using two pharmacological inhibitors^38^. We verified that Lta4h deficiency (Fig. 8d) or pharmacological inhibition of Alox5 (Fig. 8e and Supplementary Fig. 12C) were able to phenocopy the effects of cPla2 inhibition. To further confirm the above results, inhibition of cPla2 at 24 hpi, to allow the recruitment of neutrophils to the infection site, mimicked the effects of Ptgs inhibition (Fig. 8f and Supplementary Fig. 12D), while inhibition of Ptgs1/2 (Fig. 8g) and Ptgs2 (Supplementary Fig. 12E) or exogenous addition of AA (Supplementary Fig. 13B) at 24 hpi rescued the resistance of Gbp4-deficient larvae but had no effect on their WT siblings. In sharp contrast, inhibition of Alox5 (Fig. 8h and Supplementary Fig. 12F) and exogenous addition of LTB4 (Supplementary Fig. 13C) at 24 hpi did not affect larval resistance to ST.

These results suggest, therefore, the requirement of two sequential waves of eicosanoids for the clearance of bacterial infections: inflammasome-independent LT production contributes to neutrophil recruitment and then Gbp4 inflammasome-dependent PG biosynthesis is then required for neutrophil-mediated bacterial clearance. To confirm this hypothesis, we used liquid chromatography–tandem mass spectrometry (LC–MS/MS) lipidomic analysis. While eicosanoid levels in whole extracts from infected larvae were hardly affected by forced expression of Gbp4 at 8 hpi (that is, the recruitment phase), forced expression of Gbp4, and to some extent of Asc, resulted in increased levels of LTB4, lipoxin B4 (LXB4), PGE2, PGD2 and PGF2α at 24 hpi (that is, the bacterial clearance phase) (Supplementary Table 1). Curiously, however, LXA4 levels were drastically reduced by forced expression of Gbp4 or Asc at 24 hpi (Supplementary Table 1). In addition, pharmacological inhibition of caspase-1 impaired the biosynthesis of all eicosanoids in response to ST infection (Supplementary Table 2). As expected, indomethacin treatment decreased PGs to basal levels in infected larvae at 24 hpi: 0.4 pg per 1,000 larvae of PDG2, 0.3 pg per 1,000 larvae of PGE2 and 0.1 pg per 1,000 larvae of PGF2α.

**Figure 9:**
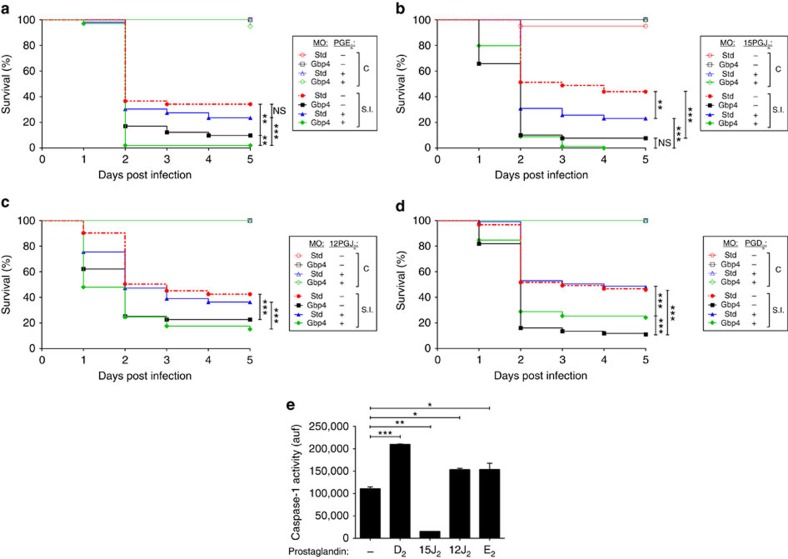
Gbp4-mediated *S.* Typhimurium clearance *in vivo* is associated to the inflammasome-dependent production of prostaglandin D2. (**a**–**e**) Zebrafish one-cell embryos were injected with standard control (std) or Gbp4 MOs, treated by immersion with vehicle alone (methyl acetate), 10 μM 16, 16-dimethyl-prostaglandin E2 (PGE_2_) (**a**), 10 μM 15-deoxy-delta-12,14-prostaglandin J2 (15PGJ_2_) (**b**), 10 μM 12-prostaglandin J2 (12PGJ_2_) (**c**), or 16,16-dimethyl-PGD2 (**d**), infected at 2 dpf (**a**–**d**), and survival (**a**–**d**) and caspase-1 activity (**e**) determined as described in Fig. 2a,b. The sample size for each treatment is 310 in **a**–**d**, 30 in **e**. auf, arbitrary units of fluorescence; NS, not significant; SI, ST infection; *P<0.05; **P<0.01; ***P<0.001 according to log-rank test (in **a**–**d**) or ANOVA followed by Tukey multiple range test (**e**).

We then analysed the impact of exogenous addition of PGE2, PGD2 and its two main derivatives 15-Deoxy-Δ12,14-PGJ2 (15dPGJ2) and Δ^12^-PGJ2 (12PGJ2) in the resistance of larvae to ST infection. While PGE2 (Fig. 9a), 15dPGJ2 (Fig. 9b) and 12PGJ2 (Fig. 9c) further increased or had no effect on the susceptibility to ST infection in both WT and Gbp4-deficient larvae, PGD2 partially rescued the higher susceptibility of Gbp4-deficient larvae, while it had no effect on their WT siblings (Fig. 9d). Notably, PGD2 was able to activate basal caspase-1 activity in zebrafish larvae, while 15dPGJ2 drastically inhibited it (Fig. 9e). Collectively, these results demonstrate (i) the critical role of Gbp4 in the inflammasome-mediated induction of PG biosynthesis and the clearance of intracellular bacteria by neutrophils, and (ii) the existence of a complex crosstalk between inflammasome and PGs *in vivo*.

## Discussion

We discovered here a novel role of GBPs in the inflammasome-dependent biosynthesis of PGs and the clearance of intracellular bacteria by neutrophils in zebrafish. Gbp4 seems to be required for neutrophil homeostasis and although this observation deserves further investigation, the critical role of Gbp4 in the control of ST infection is independent of this effect, since (i) forced expression of WT Gbp4 did not affect neutrophil numbers, while increasing inflammasome-dependent infection resistance, (ii) Gcsfa overexpression failed to reverse the high susceptibility of Gbp4-deficient larvae to ST and (iii) fish overexpressing WT Gbp4 in neutrophils showed high resistance to ST infection. The key role played by neutrophils in ST resistance in zebrafish larvae is further supported by the high resistance of larvae forced to express Gcsfa, and the failure of forced expression of both Gbp4 and Asc to increase bacterial resistance of animals deficient in Cxcr2 and Lta4h, which mediate neutrophil but not macrophage recruitment to sterile wound, bacterial pathogens, including ST, and transformed cells^27^,^35^,^36^,^39^,^40^,^41^,^42^. The role of neutrophils and macrophages in the clearance of intracellular bacteria *in vivo* is complex, probably reflecting the heterogeneous nature of the interactions between intracellular pathogens and host innate immune cells^43^. While an earlier study showed that NLRC4-dependent pyroptotic cell death of macrophages releases the intracellular pathogens into the extracellular environment rendering them susceptible to neutrophil-mediated destruction^6^, very recent studies have elegantly demonstrated that neutrophils are crucial for the clearance of ST by inflammasome-independent mechanisms, that is, oxidative stress generated through NADPH oxidase and myeloperoxidase^44^,^45^. In addition, a recent study has shown that these neutrophils are able to sustain IL-1β production during acute ST infection due to their unique resistance to pyroptotic cell death on NLRC4 inflammasome activation^46^. Similarly, infection of zebrafish larvae with a strain of *Listeria monocytogenes* overexpressing flagellin promotes inflammasome activation and macrophage, but not neutrophil, pyroptosis, which is responsible for the attenuated virulence of this strain^47^. The evolutionary conserved resistance of zebrafish neutrophils to pyroptotic cell death on strong activation of the inflammasome by ST infection and/or forced expression of Gbp4, Asc and Caspa further points out to the relevance of neutrophils in the clearance of intracellular bacterial pathogens in vertebrates.

**Figure 10:**
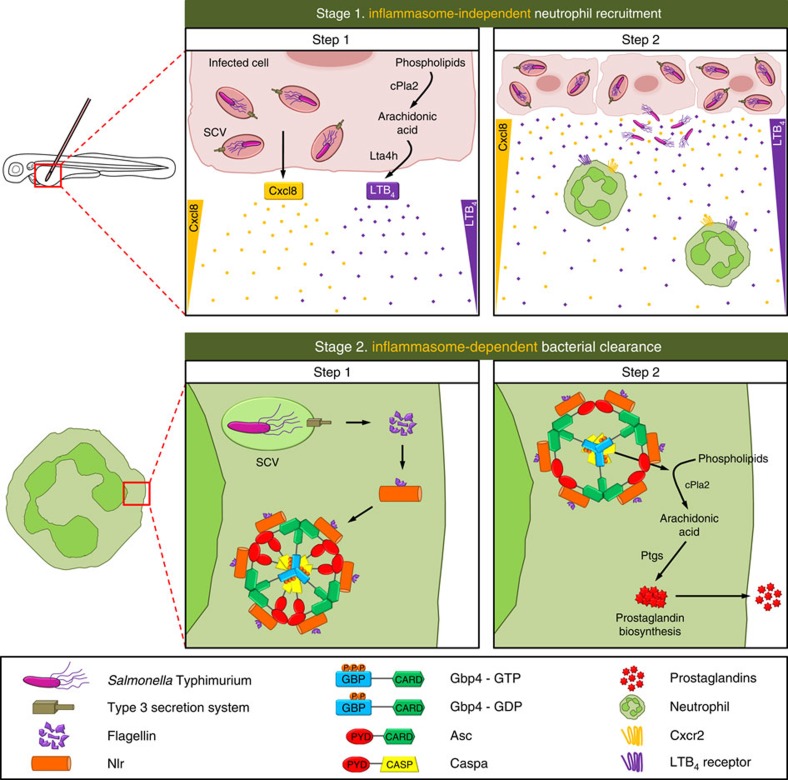
Proposed two-stage model illustrating the two sequential waves of eicosanoids produced in response to *S*. Typhimurium infection. Stage 1, step1: infected cells release Cxcl8 and LTB4 in an inflammasome-independent manner. Stage 1, step 2: neutrophils are recruited to the infection sites via Cxcl8 and LTB4 gradients and phagocytose ST. Stage 2, step1: ST taken up by neutrophils localized to the *Salmonella*-containing vacuole (SCV) where it uses its T3SS to translocate bacterial proteins to the cytosol. Flagellin, being one of those bacterial proteins, can be recognized by cytosolic NLRs, probably NLRP3 and NLRC4, which consequently induce the assembly of a Gbp4 and Asc through CARD domains allowing the subsequent recruitment of pro-Caspa. Stage 2, step2: the hydrolysis of GTP to GDP or GMP by Gbp4 results in a conformational change in the inflammasome complex that allows the activation of Caspa via autocleavage, resulting in the induction of PG biosynthesis through the activation of cPla2 and via Ptgs (also known as cyclooxygenases).

Gbp4 contains a C-terminal CARD, which in principle allows direct interaction with CARD-containing targets, omitting the need for a PYD/CARD-containing Asc adaptor^15^. However, our epistasis analysis demonstrates that Gbp4-dependent clearance of ST and caspase-1 activation both require Asc and are highly dependent on bacterial flagellin. In addition, our *in vivo* results were further confirmed by reconstituting Gbp4-Asc complexes in HEK293T cells where both WT and GTPase-deficient mutant were found to form a macromolecular complex with Asc while CARD-deficient Gbp4 was unable to interact with the Asc speck and hardly rescued the high infection susceptibility of Gbp4-deficient larvae. These results are not unexpected, since NLRC4, which also has an N-terminal CARD, requires ASC for NLRC4 inflammasome speck formation, caspase-1 activation and efficient IL-1β processing^10^. Strikingly, the Gbp4-Asc macromolecular complex observed here is rather similar to the ASC complex that comprises NLRC4, NLRP3, caspase-1, caspase-8 and pro-IL-1β, which has recently been described in macrophages infected with ST (ref. 12). We propose that Gbp4 is able to act as a novel inflammasome to recruit Asc through its CARD domain and to activate Caspa *via* its PYD domain (Fig. 10).

Another interesting, but unexpected, observation of this study is the ability of the GTPase-deficient Gbp4, but not mouse GBP5, to behave as DN, despite being able to localize with Asc specks. Although ASC assembly is abolished in tetramerization GBP5 mutants, suggesting that tetrameric GBP5 promotes Asc oligomerization, both WT and GTPase-deficient mutant GBP5 seem to form tetramers and are equally able to promote Asc multimerization^15^,^48^. This is consistent with our *in vivo* study where forced expression of mouse GTPase-deficient GBP5 did not exert a DN effect on bacterial resistance and caspase-1 activity and WT GBP5 was able to rescue bacterial susceptibility and caspase-1 activity of Gbp4-deficient larvae. These results suggest that Gbp4 would interact with a putative NLR sensor, which remains to be identified, likely though its GTPase domain, and Asc through its CARD, while GBP5 requires the interaction with the PYD domain of NLRP3 through its GTPase domain^15^. Alternatively, Gbp4 may directly sense ST infection and activates Caspa through the adaptor protein Asc. More importantly, the GTPase activity of Gbp4 is dispensable for inducing the oligomerization of Asc, like mammalian GBP5, but crucial for the subsequent activation of Caspa. Therefore, we propose a two step model where (i) the presence of flagellin in the cytosol promotes the interaction of Gbp4 with Asc through CARD domains and (ii) the hydrolysis of GTP by Gbp4 results in a conformational change in this complex that allows the oligomerization and activation of Caspa (Fig. 10).

Although it is widely accepted that inflammasome activation promotes bacterial pathogen clearance through the processing of pro-inflammatory cytokines IL-1β and IL-18 (refs 45, 49, 50, 51) and the induction of pyroptotic cell death of macrophages^6^,^52^, the relative contribution of each pathway has been a matter of controversy. In addition, some studies have even shown that strategies that inhibit inflammasome activation or downstream cytokine signalling resulted in enhanced bacterial clearance and diminished pathology^53^. We have not found any evidence of neutrophil cell death during infection of larvae with ST, being this in agreement with a recent study reporting that neutrophils do not undergo pyroptotic cell death on NLRC4 and caspase-1 activation^46^. Furthermore, although we failed to detect mature IL-1β in whole larvae, the results obtained with the luciferase-based reporter assay iGLuc (ref. 33) and the dispensability of IL-1β in ST clearance do not support a role for Asc or Caspa in the processing of zebrafish IL-1β *in vivo*, supporting the caspase-1 independent processing of IL-1β in fish macrophages^28^ and the absence of a conserved caspase-1 processing site in non-mammalian vertebrate IL-1βs (refs 29, 30). Nevertheless, our data strongly support that Gbp4 inflammasome-mediated resistance to ST is associated to the production of PGs, in agreement with the ability of NLRC4 inflammasome to generate a storm of eicosanoids in resident mouse peritoneal macrophages through the activation of cytosolic PLA2 (ref. 7). Although a particular cocktail of PGs likely favours bacterial clearance, PGD2 seems to be particularly important in the resistance to ST, while 15dPGJ2, and to some extend PGE2, had a negative effect on bacterial resistance. Curiously, 15dPGJ2 has been recently shown to inhibit caspase-1 activation in mouse macrophages through a still unknown mechanism^54^. Similarly, we also observed that 15dPGJ2 strongly inhibits caspase-1 activity *in vivo*, which might explain its negative effect on bacterial clearance. Whatever the outcome, the differential modulation of different eicosanoids, such as LXs, by the inflammasome guaranteed further investigation.

The relevance of PGs in particular, and eicosanoids in general, in the clearance of intracellular bacteria has received little attention, despite that a metabolomic study has recently shown that ST infection disturbs eicosanoid metabolism in mice^55^. Based on these *in vivo* data, the same authors performed an *in vitro* study and found that exogenous addition of 15dPGJ2 reduces macrophage colonization by ST, reactive nitrogen species production and the expression of gene encoding both pro- and anti-inflammatory cytokines^56^, and therefore making difficult to predict the impact of this PG *in vivo*. Importantly, pharmacological inhibition of PG synthesis only affected the infection resistance of larvae forced to express Gbp4 but not of those forced to express Asc. This result, although unexpected and difficult to explain, was confirmed by (i) inhibition of COX2 with meloxicam; (ii) inhibition of PLA2 with ACA and CAY10502 at 24 hpi to allow neutrophil recruitment and, therefore, leave intact LTB4-dependent neutrophil recruitment; (iii) the ability of PGD2 to reverse the susceptibility of Gbp4-deficient animal, but not of their WT siblings; and (iv) the ability of AA to increase the resistance to infection of both WT and Gbp4-deficient larvae when added at the infection time but only to increase resistance of Gbp4-deficient larvae when added 24 hpi. We speculate, therefore, that targeting eicosanoid biosynthesis may ameliorate the autoinflammatory disorders of patients with activating mutations in inflammasome components^57^,^58^ and facilitate the clearance of pathogenic bacteria able to inhibit inflammasome activation^59^.

Another interesting observation of this study is that forced expression of Gbp4 had more profound effects on eicosanoid biosynthesis than Asc, and that Pla2 activation was potentiated by forced expression of Gbp4 and Caspa, but not of Asc, in ST-infected animals. We speculate, therefore, that Gbp4 in zebrafish, and likely GBP5 or other GBPs in mammals, are required for the inflammasome-mediated regulation of PG biosynthesis. Furthermore, our genetic and pharmacological approaches have also revealed the requirement of two sequential waves of eicosanoids for the neutrophil-mediated clearance of bacterial infection in zebrafish. Hence, while inflammasome-independent LTB4 biosynthesis is first required for neutrophil recruitment to the infection site, Gbp4 inflammasome-dependent production of PGD2 mediates bacterial clearance by these cells (Fig. 10).

In summary, we report here a unique model to study the impact of inflammasome in inflammation and infectious disease progression with clear complementarities with the mouse model, among others the possibility to study the local inflammation activation and pathogen responses simultaneously in a whole organism, and the possibility of genetic and chemical screenings. Using this model, we uncovered a previously unappreciated role of neutrophils in the clearance of bacterial infection *in vivo* through the Gbp4/Asc inflammasome-dependent biosynthesis of PGs and the requirement of two independent waves of eicosanoid biosynthesis for infection clearance. The increased bacterial resistance of fish forced to express Gbp4, Asc or Caspa with no apparent side effects suggest that transient activation of the inflammasome may be beneficial in certain bacterial infectious diseases.

## Methods

### Animals

Zebrafish (*Danio rerio* H.) were obtained from the Zebrafish International Resource Center and mated, staged, raised and processed as described^60^. The lines *Tg(mpx:eGFP)*^*i114*^ (ref. 17), *Tg(mpx:Gal4.VP16)*^*i222*^ (ref. 61), *Tg(lyz:dsRED)*^*nz50*^ (ref. 62) and *Tg(mpeg1:eGFP)*^*gl22*^ (ref. 18) were previously described. The experiments performed comply with the Guidelines of the European Union Council (Directive 2010/63/EU) and the Spanish RD 53/2013. Experiments and procedures were performed as approved by the Bioethical Committees of the University of Murcia (approval numbers #537/2011, #75/2014 and #216/2014).

### DNA constructs and generation of transgenics

The genes encoding zebrafish WT Gbp4, the GTPase-deficient mutant Gbp4KS→AA, Gbp4ΔCARD, the DM Gbp4KS→AA/ΔCARD, zfiGLuc and GLuc were synthesized by GenScript Corporation. The *cmv*/*sp6:gbp4*, *cmv*/*sp6:gbp4-mCherry*, *uas:gbp4* and uas:gbp4KS→AA constructs were generated by MultiSite Gateway assemblies using LR Clonase II Plus (Life Technologies) according to standard protocols and using Tol2kit vectors described previously^63^. The zebrafish Asc-Myc, Caspa and Caspb (ref. 24), zebrafish Gcsfa (ref. 64) and mouse WT GBP5 and the GTPase-deficient mutant GBP5(S52N)^15^ were previously described.

The lines *Tg(uas:gbp4)*^*ums2*^ and *Tg(uas:gbp4KS→AA)*^*ums3*^ were generated by microinjecting 0.5–1 nl into the yolk sac of one-cell-stage embryos a solution containing 100 ng μl^−1^ *uas:gbp4* and *uas:gbp4KS→AA* constructs, respectively, and 50 ng μl^−1^ Tol2 RNA in microinjection buffer (× 0.5 Tango buffer and 0.05% phenol red solution) using a microinjector (Narishige).

### Morpholino and RNA injection

Specific MOs (Gene Tools) were resuspended in nuclease-free water to 1 mM (Supplementary Table 3). *In vitro*-transcribed RNA was obtained following manufacturer's instructions (mMESSAGE mMACHINE kit, Ambion). MOs and RNA were mixed in microinjection buffer and microinjected into the yolk sac of one-cell-stage embryos using a microinjector (Narishige; 0.5–1 nl per embryo). The same amount of MOs and/or RNA were used in all experimental groups. The efficiency of the MOs was checked by PCR with reverse transcription and/or western blot.

### Chemical treatments

Two or three dpf larvae were treated by bath immersion with the Ptgs inhibitors indomethacin (10 μM, Sigma-Aldrich) and meloxicam (10 μM, Cayman Europe), the Pla2 inhibitors ACA (0.5 μM, Santa Cruz Biotechnology) and CAY10502 (5 μM, Cayman Europe), the Alox5 inhibitors MK-886 (1 μM) and Zileuton (20 μM, both from Cayman Europe), the caspase-1 inhibitor Ac-YVAD-CMK (100 μM, Peptanova) or the pan-caspase inhibitor Q-VD-OPh (50 μM, SM Biochemicals LLC), LTB4 (1 μM, Cayman Europe) and AA (20 μM, Cayman Europe) diluted in egg water supplemented with 0.5% DMSO, or with 10 μM of 16, 16 dimethyl PGE2, 16, 16 dimethyl PGD2, 12PGJ2 or 15dPGJ2 (all from Cayman Chemical) diluted in egg water supplemented with 0.01–0.2% methylacetate (Sigma-Aldrich).

### Infection assays

For most infection experiments, ST 12023 (WT) and the isogenic derivative mutants for SPI-1 (prgH020::Tn5lacZY), SPI-2 (ssaV::aphT) and SPI-1/SPI-2 (prgH020::Tn5lacZY ssaV::aphT; kindly provided by Prof. D. Holden) were used. For some experiments the ST strains used were: 14028s (WT), its isogenic derivative mutant fliC/fljB, and the FliCON, which persistently expresses the flagellin protein FliC (refs 6, 23; kindly provided by Dr E.A. Miao). For otic vesicle infections, ST 12023 expressing DsRedT3 (ref. 27) was used (ST:DsRedT3). Overnight cultures in Luria-Bertani (LB) broth were diluted 1/5 in LB with 0.3 M NaCl, incubated at 37 °C until 1.5 optical density at 600 nm was reached, and finally diluted in sterile PBS. Larvae of 2 dpf were anaesthetized in embryo medium with 0.16 mg ml^−1^ tricaine and 10 bacteria (yolk sac) or 100 (otic vesicle) per larvae were microinjected (Fig. 2a). Larvae were allowed to recover in egg water at 28–29 °C, and monitored for clinical signs of disease or mortality over 5 days. At least three independent experiments were performed with a total number of 200–350 larvae per treatment.

### Caspase-1 and Pla2 activity assays

The caspase-1 activity was determined with the fluorometric substrate Z-YVAD 7-Amido-4-trifluoromethylcoumarin (Z-YVAD-AFC, caspase-1 substrate VI, Calbiochem) as described previously^20^,^28^. In brief, 25–35 larvae were lysed in hypotonic cell lysis buffer (25 mM 4-(2-hydroxyethyl)piperazine-1-ethanesulfonic acid, 5 mM ethylene glycol-bis(2-aminoethylether)-N,N,N′,N′-tetraacetic acid, 5 mM dithiothreitol, 1:20 protease inhibitor cocktail (Sigma-Aldrich), pH 7.5) on ice for 10 min. For each reaction, 10 μg protein were incubated for 90 min at 23 °C with 50 μM YVAD-AFC and 50 μl of reaction buffer (0.2% 3-[(3-cholamidopropyl)dimethylammonio]-1-propanesulfonate (CHAPS), 0.2 M 4-(2-hydroxyethyl) piperazine-1-ethanesulfonic acid, 20% sucrose, 29 mM dithiothreitol, pH 7.5). After the incubation, the fluorescence of the AFC released from the Z-YVAD-AFC substrate was measured with a FLUOstart spectofluorometer (BGM, LabTechnologies) at an excitation wavelength of 405 nm and an emission wavelength of 492 nm (Fig. 2b). A representative caspase-1 activity assay out of three is shown accompanying each survival assay.

Pla2 activity was determined in 50 μg protein extracts obtained from whole-zebrafish larvae using the EnzChek Phospholipase A2 Assay Kit (ThermoFisher Scientific) following the manufacturer's recommendations. The data were normalized using extracts treated with 0.5 μM ACA.

### Cell sorting

Approximately 300–500 larvae were anaesthetized in tricaine, minced with a razor blade, incubated at 28 °C for 30 min with 0.077 mg ml^−1^ Liberase (Roche) and the resulting cell suspension passed through a 40-μm cell strainer. Cell sorting was performed on a FACSCalibur (BD Biosciences) and a SH800Z (Sony).

### Neutrophil recruitment and pyroptotic cell death assays

To study neutrophil recruitment and pyroptotic cell death in a localized site of ST infection, 2 dpf *mpx:eGFP* larvae were anaesthetized in embryo medium with 0.16 mg ml^−1^ tricaine and mounted in 1% low melting point agarose supplemented with 0.16 mg ml^−1^ tricaine. PBS (0.5 nl) or ST:DsREDT3 suspension (100 bacteria/larva), supplemented with phenol red, was then injected into the otic ear vesicle. Embryo medium with 0.16 mg ml^−1^ tricaine solution was added, to maintain the embryos hydrated during the experiments. Images of the otic area were taken at 1 and 24 post-infection (hpi) using a Leica MZ16F fluorescence stereo microscope, treated with ImageJ software (http://rsb.info.nih.gov/ij/) and neutrophil counts determined. For the analysis of pyroptotic cell death, the experiments were performed as above but using *lyz:dsRED* larvae, non-fluorescent ST and 2 μM of the cell-impermeable green fluorescent dye YO-PRO (Life Technologies) that was injected in the otic vesicle at 3 hpi. Images were taken at 4.5 and 24 hpi.

### Luminiscence

Embryos were microinjected as described above with 200 pg of *in vitro*-transcribed zfiGLuc or GLuc RNA. After 48 h, larvae were infected with WT ST (MOI of 10) and whole-larval extracts were obtained at 24 hpi as described previously^65^. Larval extracts were then combined 1:1 with distilled water containing 4.4 μM coelenterazine (Sigma-Aldrich) to achieve a final concentration of 2.2 μM. The luciferase signal was then measured on a Luminometer Optocomp II (MGM Instruments).

### Analysis of gene expression

Total RNA was extracted from whole embryos/larvae or sorted cells with TRIzol reagent (Invitrogen) following the manufacturer's instructions and treated with DNase I, amplification grade (1 U μg^−1^ RNA; Invitrogen). SuperScript III RNase H^−^ Reverse Transcriptase (Invitrogen) was used to synthesize first-strand complementary DNA with oligo(dT)18 primer from 1 μg of total RNA (10–100ng for sorted cells) at 50 °C for 50 min. Real-time PCR was performed with an ABI PRISM 7500 instrument (Applied Biosystems) using SYBR Green PCR Core Reagents (Applied Biosystems). Reaction mixtures were incubated for 10 min at 95 °C, followed by 40 cycles of 15 s at 95 °C, 1 min at 60 °C, and finally 15 s at 95 °C, 1 min 60 °C and 15 s at 95 °C. For each mRNA, gene expression was normalized to the ribosomal protein S11 (*rps11)* content in each sample using the Pfaffl method^66^. The primers used are shown in Supplementary Table 4. In all cases, each PCR was performed with triplicate samples and repeated at least with two independent samples.

### Inflammasome reconstitution in HEK293T cells and immunofluorescence

HEK293T cells (CRL-11268; American Type Culture Collection) were maintained DMEM:F12 (1:1) supplemented with 10% FCS, 2 mM Glutamax and 1% penicillin–streptomycin (Life Technologies). Plasmid DNA was prepared using the Mini-Prep procedure (Qiagen). DNA pellets were resuspended in water and further diluted, when required, in PBS. Transfections were performed with a cationic lipid-based transfection reagent (LyoVec, Invivogen) according to the manufacturer's instructions. Briefly, HEK293T cells were plated in 24-well plates (120,000 cells per well) together with 25 μl transfection reagent containing a total of 250 ng of total plasmid DNA. The Gpb4-GFP and the Asc-myc constructs were used at a ratio of 1:1. For immunofluorescence assays, HEK293T cells were seeded on poly-L-lysine coated coverslips, washed twice with PBS, fixed with 4% formaldehyde in PBS for 15 min at room temperature and then washed three times with PBS. Cells were then blocked with 1% bovine serum albumin (BSA, Sigma-Aldrich) and cells were permeabilized with 0.2% saponin (Fluka) in PBS for 30 min at room temperature. After that, cells were incubated for 1 h at room temperature with a mouse monoclonal antibody against c-myc (1:1,000 dilution; 46-0603; Invitrogen). Cells were washed and then were incubated for 1 h at room temperature with Alexa Fluor 647 donkey anti-mouse IgG (1:200 dilution; A-31571; Life technologies), then were rinsed in PBS and mounted on slides with ProLong Diamond Antifade Mountant with DAPI (Life Technologies). Images were acquired with a Nikon Eclipse Ti microscope equipped with a × 60 Plan Apo Vc objective (numerical aperture, 1.40) and a digital Sight DS-QiMc camera (Nikon) with a Z optical spacing of 0.2 μm and 387-nm/447-nm, 472-nm/520-nm and 650-nm/668-nm filter sets (Semrock). Images were deconvolved using ImageJ software, and maximum-intensity projections of deconvolved images are shown in the results.

### Western blot assays

The protein concentrations of cell lysates were estimated by the bicinchoninic acid (BCA) protein assay reagent (Pierce) using BSA as a standard. Cell extracts from HEK293T cells (60 μg) or dechorionated and deyolked larvae at 3 dpf obtained using 200 μl lysis buffer (10 mM Tris-HCl pH 7.4, 150 mM NaCl, 1% Triton X-100, 0.5% NP-40 and a 1:20 dilution of the protease inhibitor cocktail P8340 from Sigma-Aldrich) were resolved on 10% SDS-PAGE, transferred for 50 min at 200 mA to nitrocellulose membranes (BioRad), probed with a 1/200 dilution of four different mAbs to zebrafish Gbp4 generated using the SEAL technology (Abmart; Supplementary Fig. 1), with a 1/5,000 dilution of the mouse monoclonal Ab against c-myc (46-0603; Invitrogen) or 1/1,000 dilution of the rabbit polyclonal anti-GFP (sc-8334; Santa Cruz), and developed with adequate HRP labelled secondary antibodies or with enhanced chemiluminescence (ECL) reagents (GE Healthcare) according to the manufacturer's protocol. In some experiments, membranes were then reprobed with a 1/5,000 dilution of a commercial rabbit antibody to histone 3 (#ab1791, Abcam), as an appropriate loading control. Images have been cropped for presentation in Figs 1 and 6. Full size images are presented in Supplementary Fig. 14.

### Eicosanoid determination

One thousand larvae were collected at 3 dpf and snap frozen in liquid nitrogen. Eicosanoid were extracted and analysed by LC–MS/MS as previously described^67^,^68^.

### Statistical analysis

Data are shown as mean±s.e.m. and were analysed by analysis of variance and a Tukey multiple range test to determine differences between groups. The differences between two samples were analysed by the Student's *t*-test. A log-rank test was used to calculate the statistical differences in the survival of the different experimental groups.

### Data availability statement

All relevant data are available from the authors on request and/or are included with the manuscript (as figure source data or Supplementary information files).

## Additional information

**How to cite this article**: Tyrkalska, S.-D. *et al.* Neutrophils mediate *Salmonella* Typhimurium clearance through the GBP4 inflammasome-dependent production of prostaglandins. *Nat. Commun.* 7:12077 doi: 10.1038/ncomms12077 (2016).

## Supplementary Material

Supplementary Information Supplementary Figures 1-14 and Supplementary Tables 1-4
