# Supplementary material for: Neutrophils mediate Salmonella Typhimurium clearance through the GBP4 inflammasome-dependent production of prostaglandins
Source: Nat Commun. 2016 Jul 1;7:12077. doi: 10.1038/ncomms12077 (PMC4932187; doi:10.1038/ncomms12077)
Supplement: Supplementary Information — Supplementary Figures 1-14 and Supplementary Tables 1-4 [file ncomms12077-s1.pdf]

MDKPVCLIDTGSDGKLCVQQAALQVLQQIQQPVVVVAVVGLYRTG**KS**FLMNRLAG 55

KRTGFALSSNIKPKTEGIWMWCVPHPTKAGTSLVLLDTKGLGDVEKGDSKRDTYI 110

FSLTVLLSSTLVYNSRGVIDNKAMEELQYVTELIEHIKVTPDEDADDCTAFAKFF 165

PHFIWCLRDFTLELKLDGKDLTEDEYLEFALKLRPGTLKKVMMYNLPRECIQKFF 220

PCRTCFTFPSPTTPEKRSILESLSPAELDPEFLEVTKRFCKFVFDRSEVKQLKGG 275

HTVTGRVLGNLTKMYVDTISSGAVFCLENAVIAMAQIENEAATQEGLEVYQRGME 330

KLKSSFPLELEQVSSEHQRLSRMATQAFMARSFKDTDGKHLKALEGEMGKLFDAY 385

RSQNKQVGLETHCDLLLYMRCKDPLILHVYFFPVNDARSKEKVEQNERSSLPISH 440

PRPDRPFQVKTPHVLEVPGASVYPEEGISFRTDVEPNFFKVRKLQVDDVQMNLVR 495

QKDKMSVWTTTIWKEEFVHLQQVRDERKLNSEIEKNDFFNAHRVAFIERVTNVKS 550

IADKLHGQRIIHKELYSEITQTNVTRQQIMRKICDSVDSSGRIAKCKFIDILQEE 605

ERCLLEDLKLSES 618

Ab1 (57-68) **Ab2** (235-247) **Ab3** (364-375) **Ab4** (567-578)

GBP-N domain GBP-C domain CARD domain

Supplementary Figure 1, related to Figure 1. Epitopes recognized by the four monoclonal antibodies against zebrafish Gbp4. Ab1 and Ab2 bind to the GBP, N-terminal domain (yellow), Ab3 to the GBP, C-terminal (grey), and Ab4 to the CARD domain (black). Binding sites are shown in different colours. The essential residues of the nucleotide-binding pocket that have been mutated to obtain the GTPase-deficient mutant are in bold and red font.

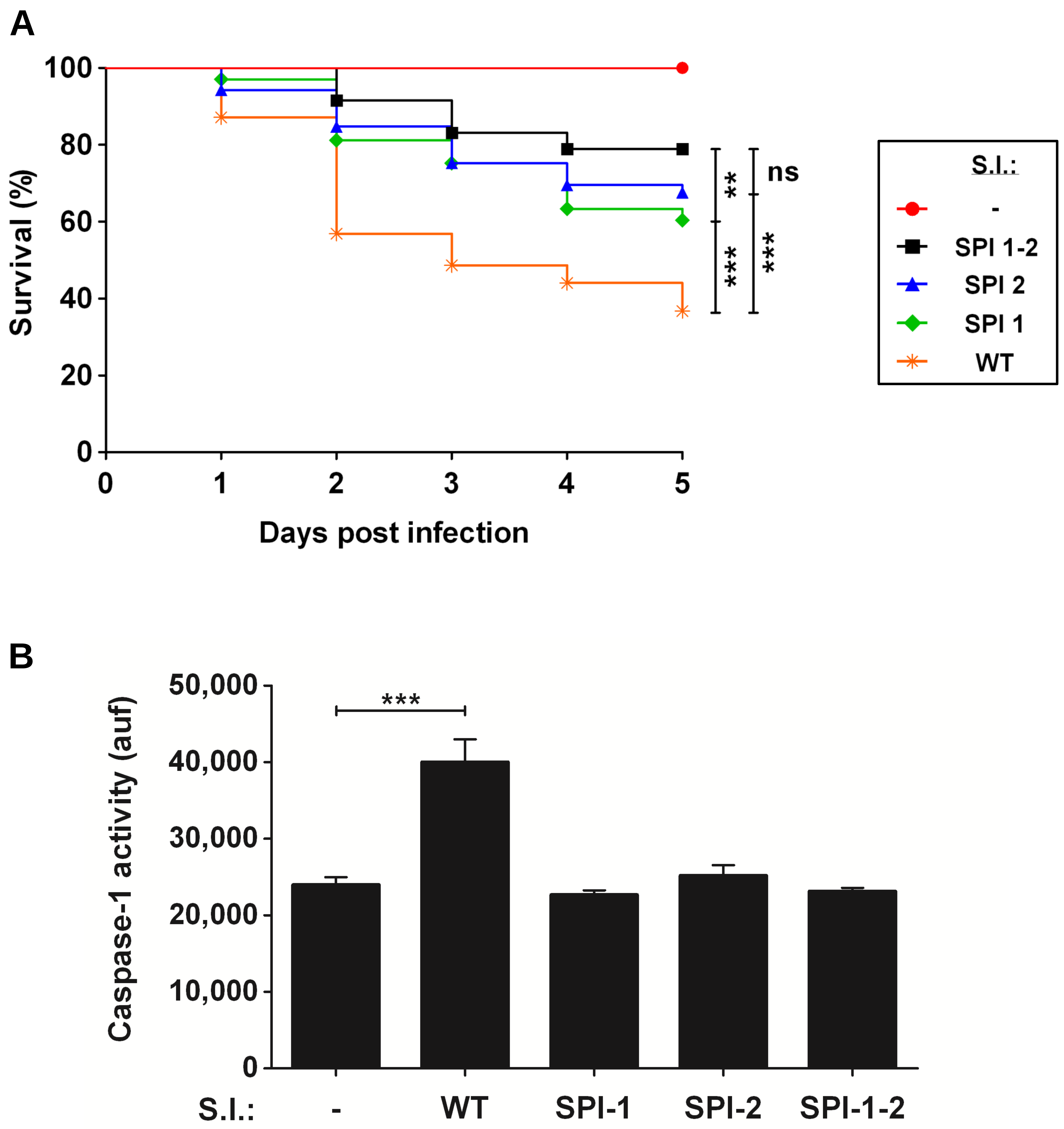

Supplementary Figure 2, related to Figure 2. The T3SS of *S*. Typhimurium is required for its virulence in zebrafish. Survival (A) and caspase-1 activity (B) were determined as described in Figures 2A and 2B, respectively. Four different strains of ST were used: the wild type (WT) and its isogenic derivative mutants SPI-1, SPI-2 and SPI-1-2. The sample size for each treatment is 280 in A, 30 in B. S.I., ST infection. ns, not significant; ***p*<0.01; ****p*<0.001 according to log rank test (A) or ANOVA followed Tukey multiple range test (B).

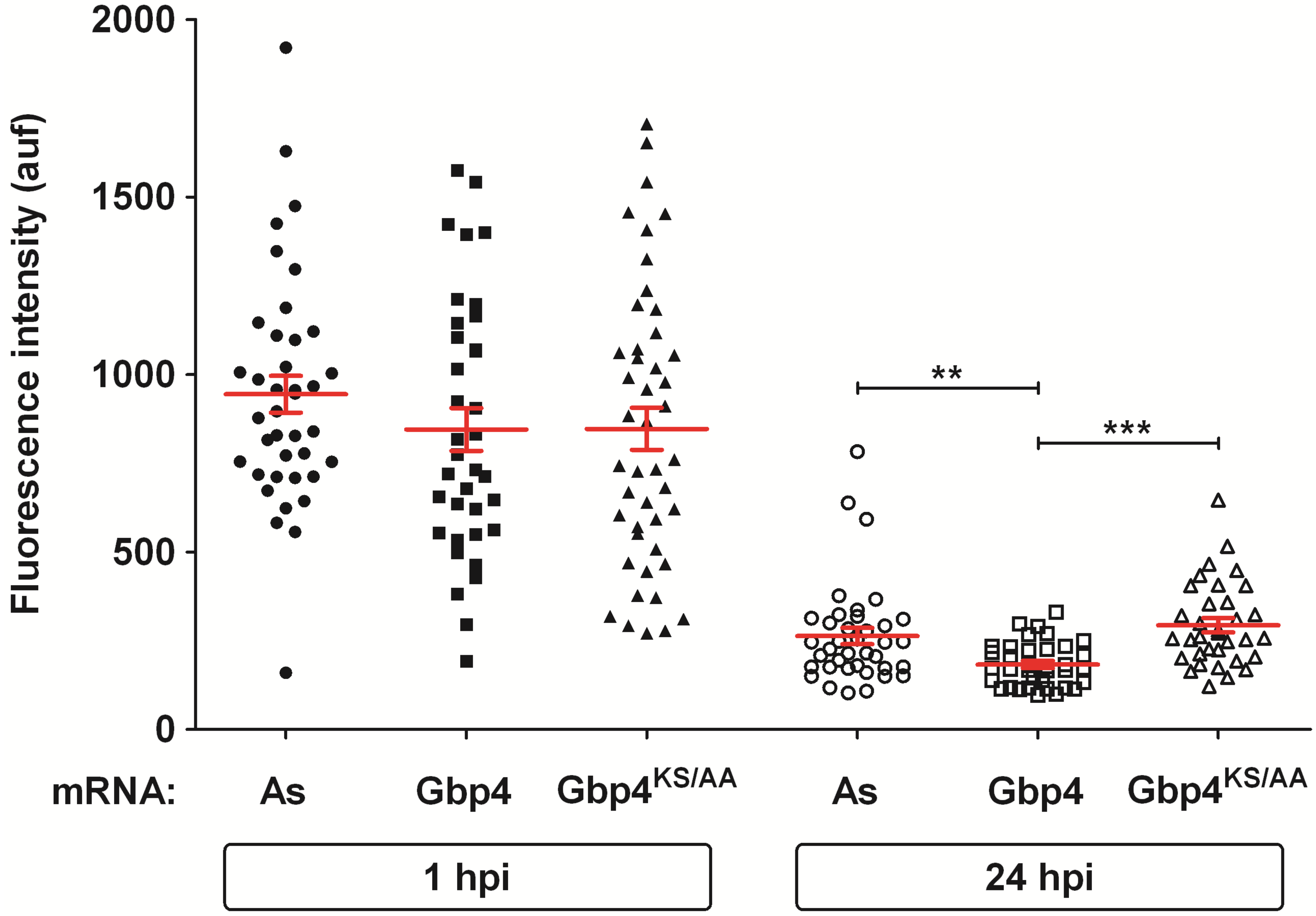

Supplementary Figure 3, related to Figures 2 and 4: Gbp4 regulates *S*. Typhimurium clearance. Zebrafish one-cell stage embryos were microinjected with antisense (As), Gbp4 or Gbp4$^{KS/AA}$ mRNAs and infected at 2 dpf with ST:DsRedT3 into the otic vesicle. Fluorescence intensity quantification of ST:DsRedT3 bacteria at 1 and 24 hpi. Each dot represents the fluorescence from a single larva (N=36, 35, 41, 36, 35, 41). The mean ± SEM of the fluorescence for each group is also shown. Scale bars = 100µm. auf, arbitrary units of fluorescence. ***p*<0.01; ****p*<0.001 according to ANOVA followed by Tukey multiple range test.

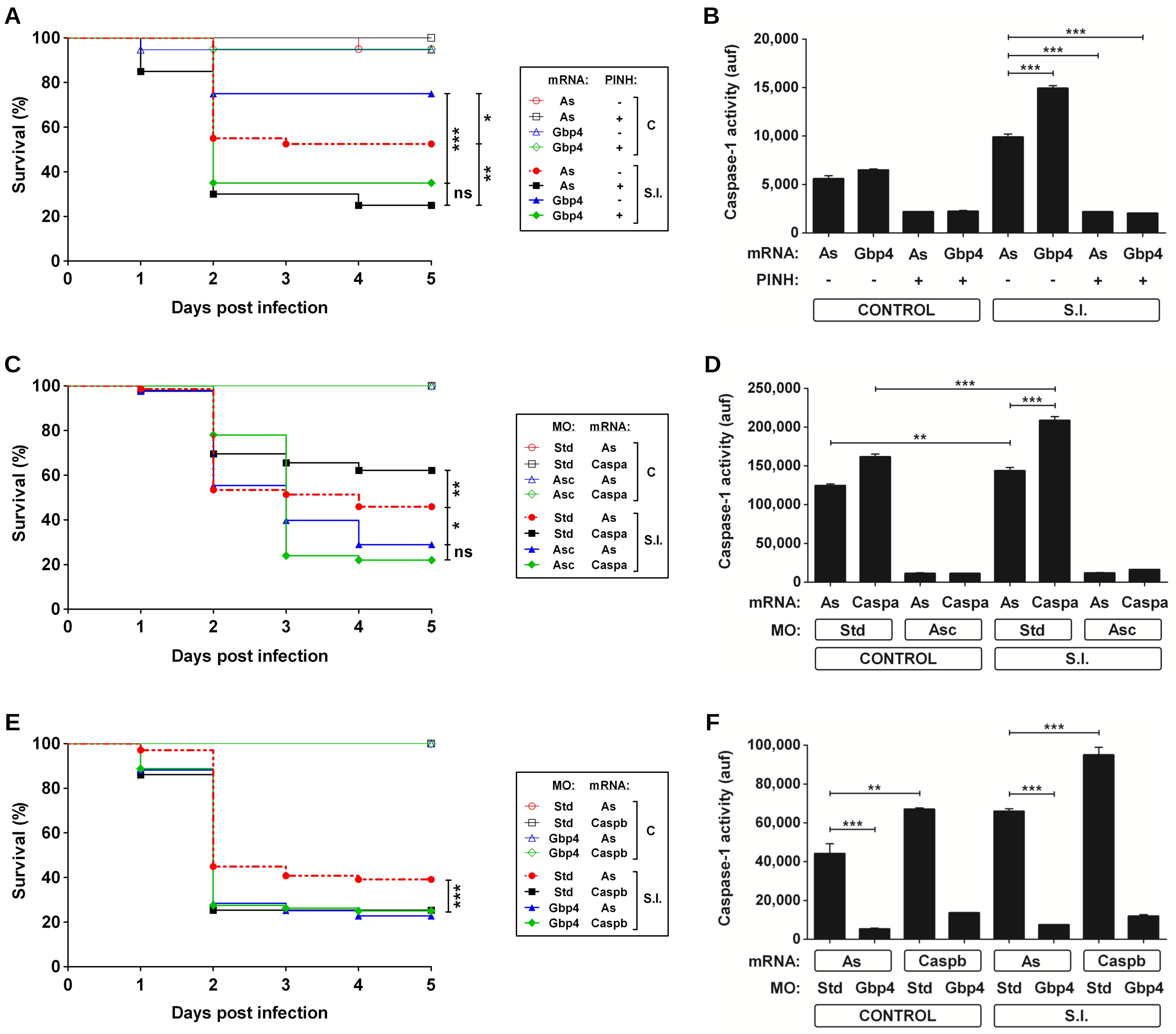

Supplementary Figure 4, related to Figure 3. The Gbp4-mediated resistance to *S.* Typhimurium is Caspa dependent. (A, B) Zebrafish one-cell embryos were injected with antisense (As) or Gbp4 mRNAs and treated by immersion with vehicle alone (DMSO) or 50 µM of a general inhibitor of caspases (Q-VD-OPh, PINH). (C, D) Zebrafish one-cell embryos were injected with standard control (Std) or Asc MOs in combination with antisense (As) or Caspa mRNAs. (E, F) Zebrafish one-cell embryos were injected with standard control (Std) or Gbp4 MOs in combination with antisense (As) or Caspb mRNAs. At 2 dpf, larvae were infected and survival (A, C, E) and caspase-1 activity (B, D, F) determined as described in Figures 2A and 2B, respectively. The sample size for each treatment is 340 in A, C and E, 30 in B, D and F. S.I., ST infection. ns, not significant; *$p<0.05$; **$p<0.01$; ***$p<0.001$ according to log rank test (A, C and E) or ANOVA followed by Tukey multiple range test (B, D and F).

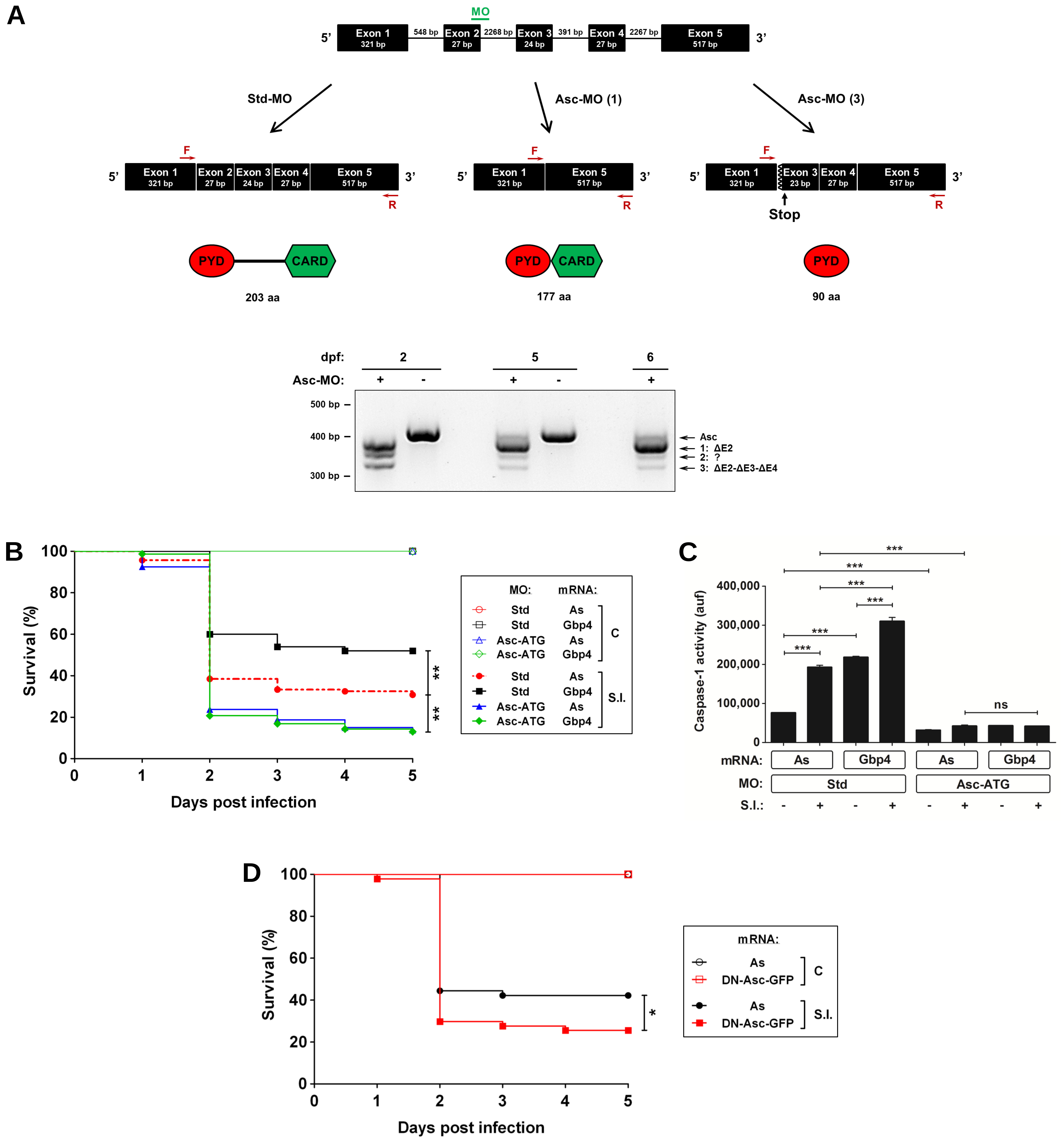

Supplementary Figure 5, related to Figure 3. Asc is crucial for *S.* Typhimurium resistance in zebrafish. (A) Schematic representations of wild type and predicted altered spliced transcripts and truncated Asc protein, and RT-PCR analysis of Asc MO-induced altered splicing of *asc* transcript at the indicated times. Samples from embryos injected with a standard control MO (Std) are shown for comparison. The primer pairs (F and R) used for amplification and the annealing of the MO are indicated with arrows and a dashed line, respectively. The altered splicing of the *asc* transcripts resulted in three smaller amplification products than the one observed in samples injected with a standard control MO. The highest product contained a complete deletion of exon 2 and the first base pair of exon 3, which resulted in a predicted truncated Asc protein containing the PYD alone and, therefore, may act as a dominant negative (DN) form [1]. The smaller transcript contained a deletion of exons 2, 3 and 4 that resulted in a predicted Asc protein lacking the linker between the PYD and CARD, which may be functional, since a spliced variant of mouse ASC lacking this linker was found to activates caspase-1 to a similar degree that the full length ASC [2]. Finally, we failed to sequence the medium size *asc* transcript but, judging from its size, it should contain a deletion of exons 2 and 3 or exons 3 and 4 that would result in Asc proteins with a shortened linker between PYD and CARD. (B, C) Zebrafish one-cell embryos were injected with standard control (Std) or Asc-ATG MOs in combination with antisense (As), GBP4 (B, C) or DN-Asc-GFP (D) mRNAs, infected at 2 dpf, and survival (B) and caspase-1 activity (C) determined as described in Figures 2A and 2B, respectively. The sample size for each treatment is 35 in A, 320 in B and D, 30 in C. S.I., ST infection. ns, not significant; *p<0.05; **p<0.01; ***p<0.001 according to log rank test (B and D) or ANOVA followed by Tukey multiple range test in C.

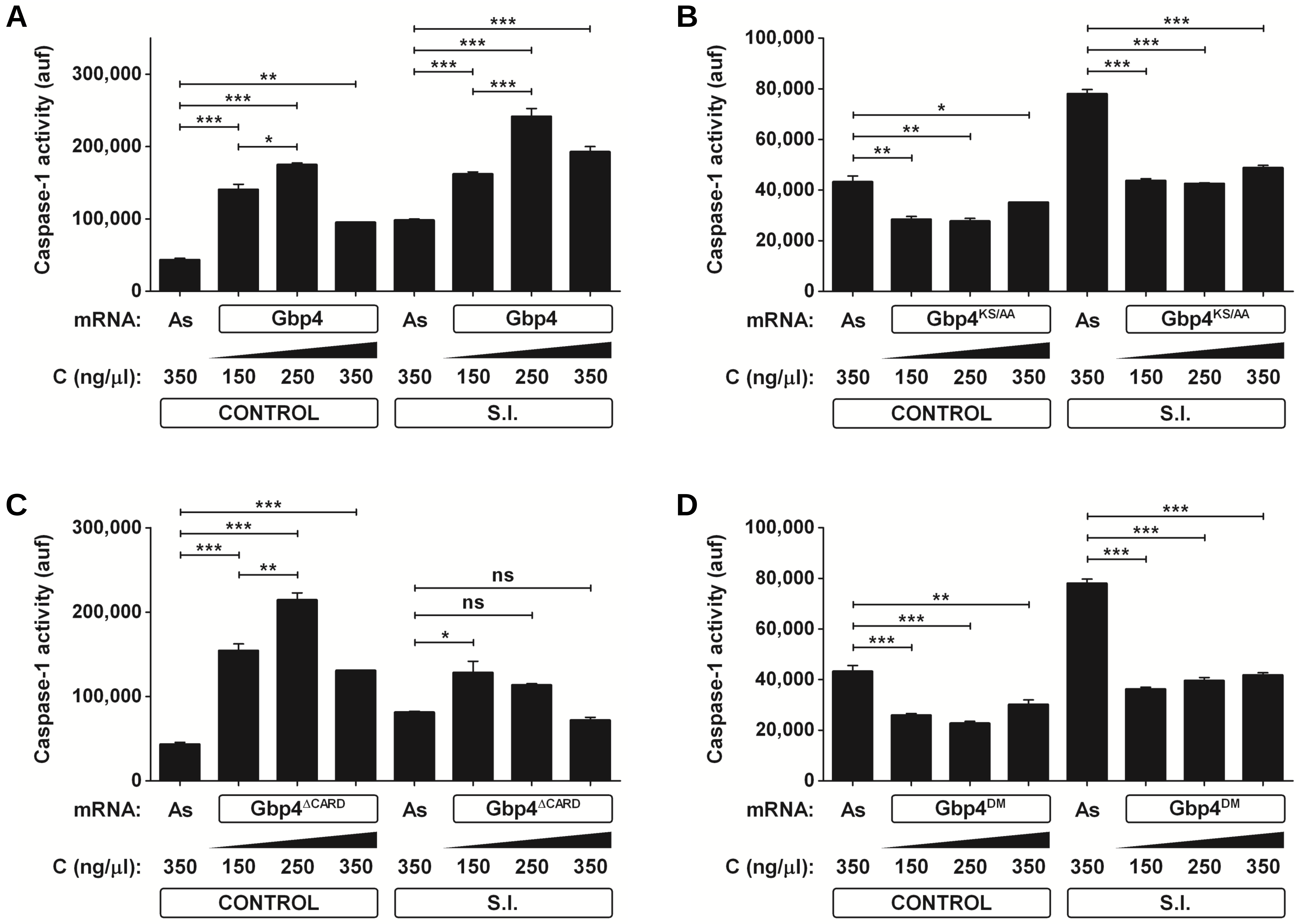

Supplementary Figure 6, related to Figure 5. Gbp4 levels fine-tune inflammasome activation and caspase-1 activity. Zebrafish one-cell embryos were injected with antisense (As) or increasing concentrations of Gbp4 (A), Gbp4$^{\Delta CARD}$ (B), Gbp4$^{KS/AA}$ (C) or Gbp4^{DM} (D) mRNAs. Caspase-1 activity was determined as described in Figure 2B. The sample size for each treatment is 30 in A-D. S.I., ST infection. ns, not significant; **p*<0.05; ***p*<0.01; ****p*<0.001 according to ANOVA followed by Tukey multiple range test (A-D).

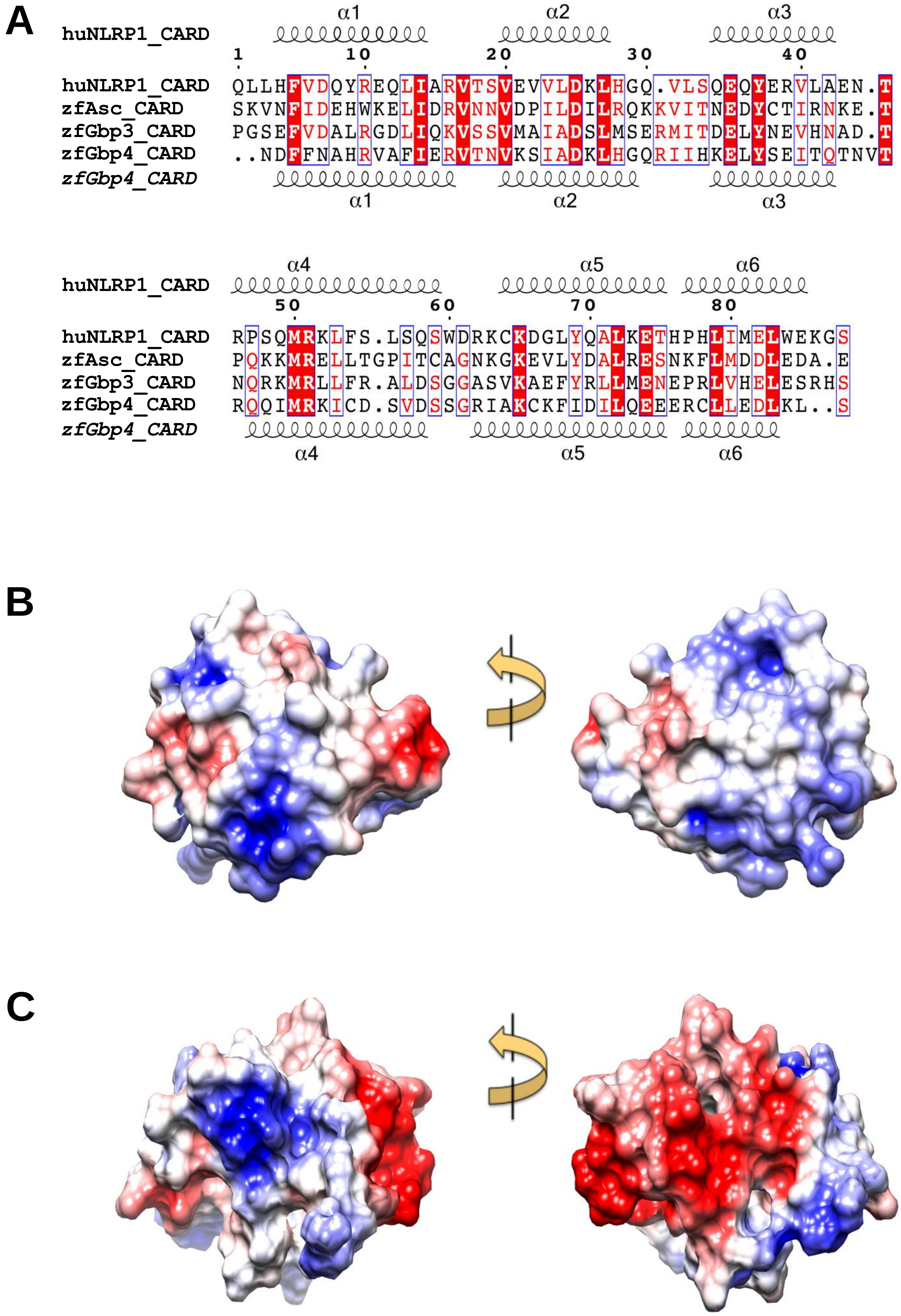

Supplementary Figure 7, related to Figure 6. Gbp4 and Asc CARD domains. (A) ESPript output [3] obtained with representative CARD domains retrieved from Uniprot database: Human NLRP1 (Q9C000, residues 1377-1466), zebrafish Asc (A8E7Q7, residues 115-203), zebrafish Gbp3 (B0V1H4, residues 870-958) and zebrafish Gbp4 (A4QNT4, residues 531-616). Residues strictly conserved are in red. Springs above or below blocks of sequences represent helices. (B, C) Electrostatic charge surface of the Gbp4-CARD and zfAsc-CARD structures. The electrostatic charge surface patches of the CARD structures obtained from homology models using Chimera [4] are displayed on a scale of -5 kT/e (red) to 5 kT/e (blue).

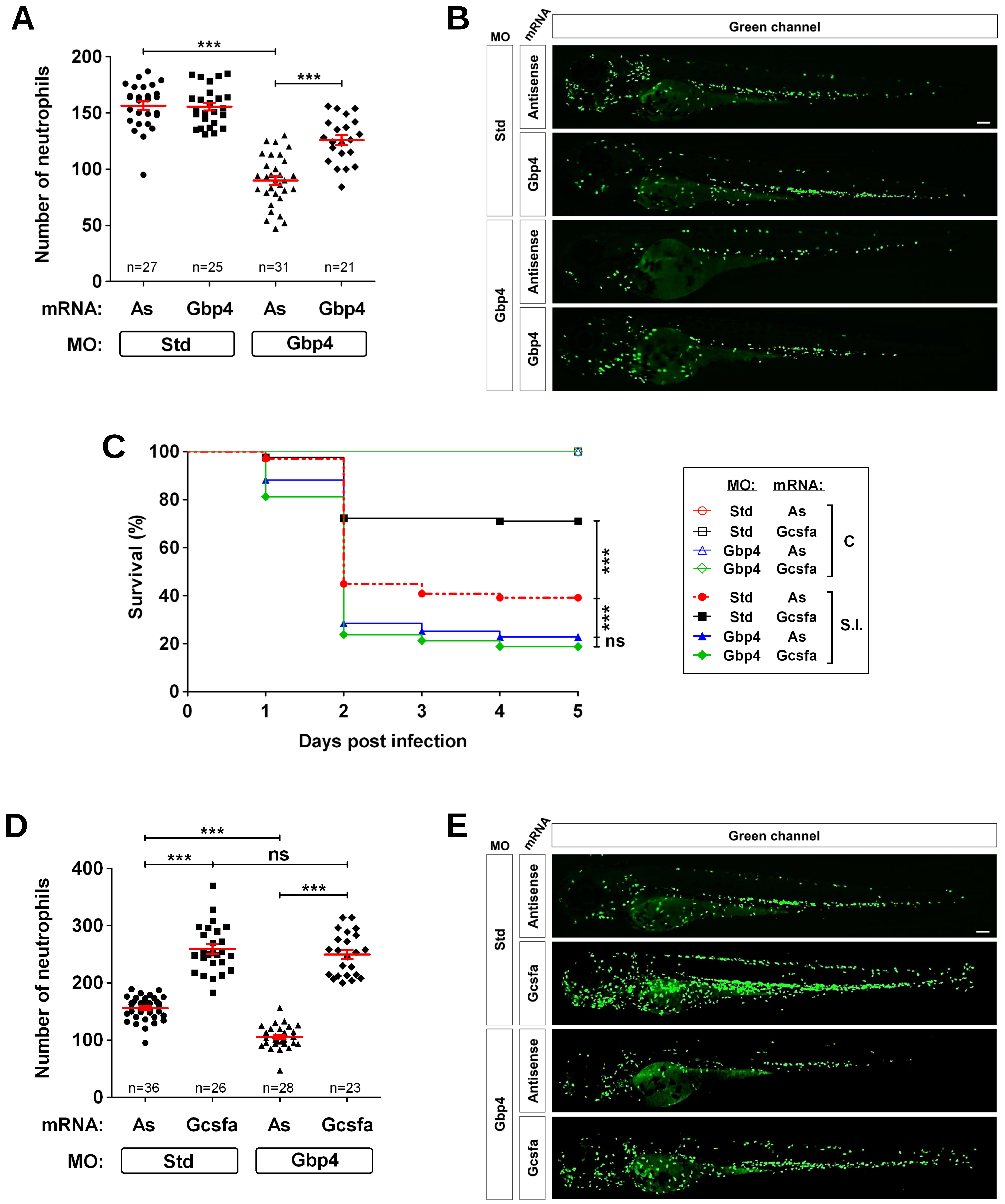

Supplementary Figure 8, related to Figure 7. Gbp4 regulates the number of neutrophils. Zebrafish *mpx:eGFP* one-cell embryos were injected with standard control (Std) or Gbp4 MOs in combination with antisense (As), Gbp4 (A-E) or Gcsfa (C-E) mRNAs (A, D). Each dot represents the number of neutrophils from a single larva, while the mean ± SEM for each group is also shown. The sample size (n) is indicated for each treatment. (C) At 2 dpf, larvae were infected and survival determined as described in Figures 2A. (B, E) Representative images of green channels of whole larvae for the different treatments. Scale bars, 100 µm. The sample size for each treatment is shown in the graph in A and D, and is 290 in C . ns, not significant; ***$p<0.001$ according to log rank test (C) or ANOVA followed by Tukey multiple range test (A and D).

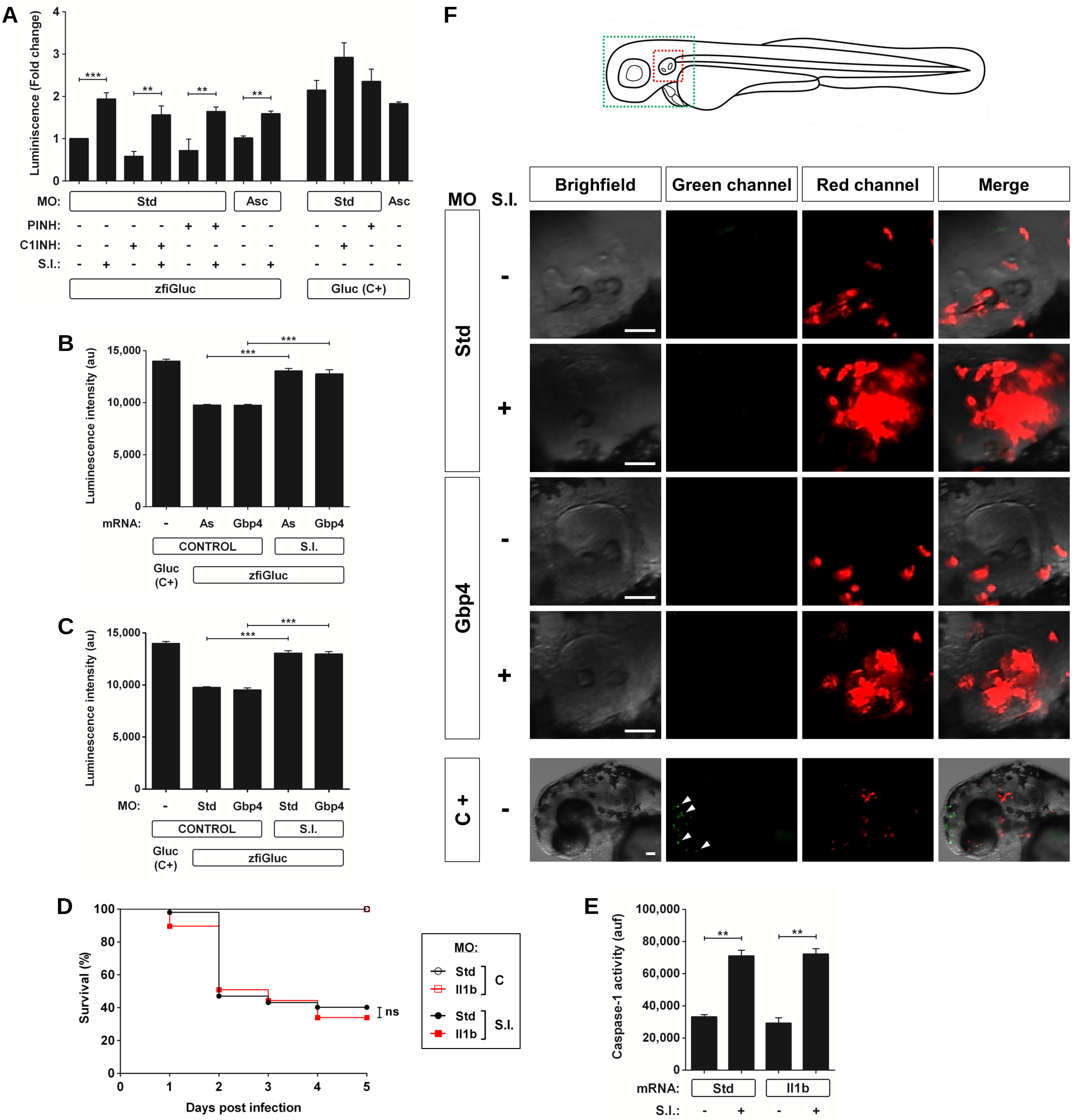

Supplementary Figure 9, related to Figures 8, 9. The Gbp4-mediated resistance to *S.* Typhimurium is independent of IL-1β processing and pyroptotic cell death. (A-E) Zebrafish one-cell embryos were injected with standard control (Std), Asc (A), Gbp4 (C), or Il1b (D, E) MOs in combination with antisense (As) or Gbp4 mRNAs (B) and zfiGluc mRNA (A-C), treated by immersion with vehicle alone (DMSO), 100 µM of a general inhibitor of caspases (PINH) or 100 µM of a specific inhibitor of caspase-1 (C1INH) (A), and infected at 2 dpi with ST (MOI of 10). The luciferase activity determined at 24 hpi as described in the Method section (A-C), while survival (D) and caspase-1 activity (E) were determined as described in Figures 2A and 2B, respectively. The Gluc was used as a positive control (C+). (F) Zebrafish *lys:dsRED* one-cell embryos were injected with standard control (Std) or Gbp4 MOs and infected at 2 dpf in the otic vesicle with ST at a MOI of 100. YO-PRO compound was injected at 3 hpi in the otic vesicle and pictures of each larva were taken at 4.5 hpi at the fluorescent microscope to visualize dead cells. Representative pictures are shown for each treatment. As a positive control, a group of cells dying during larvae development in the front part of the head are shown. The sample size for each treatment is 25 in A-C, 300 in D, 30 in E, 40 in F. S.I., ST infection. ns, not significant. **p<0.01; ***p<0.001 according to log rank test (D) or ANOVA followed by Tukey multiple range test (A, B, C and E).

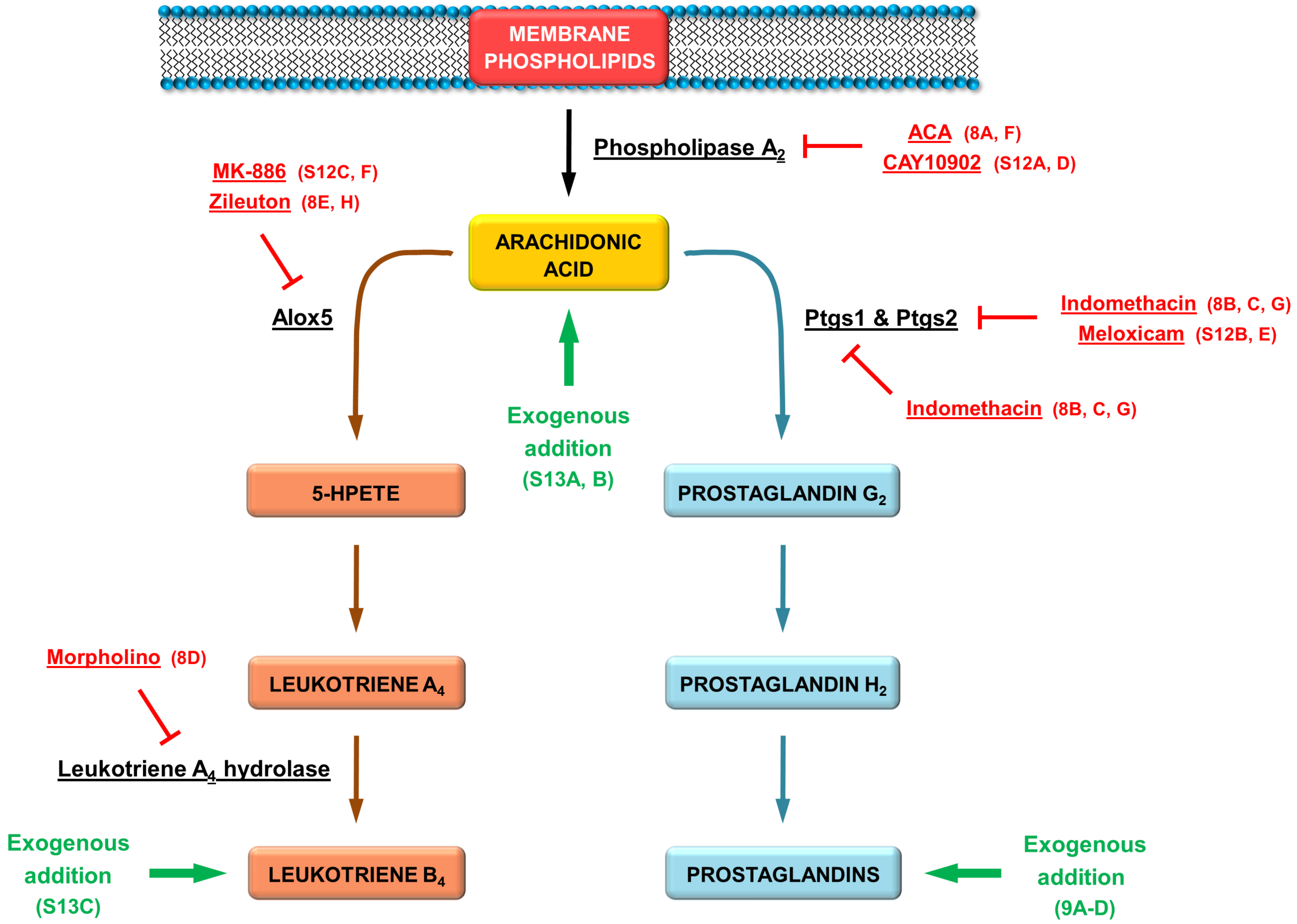

Supplementary Figure 10, related to Figures 8, Supplementary (S) 11-13. Biosynthesis of eicosanoids. The different pharmacological inhibitors and the lipid mediators used in this study are shown.

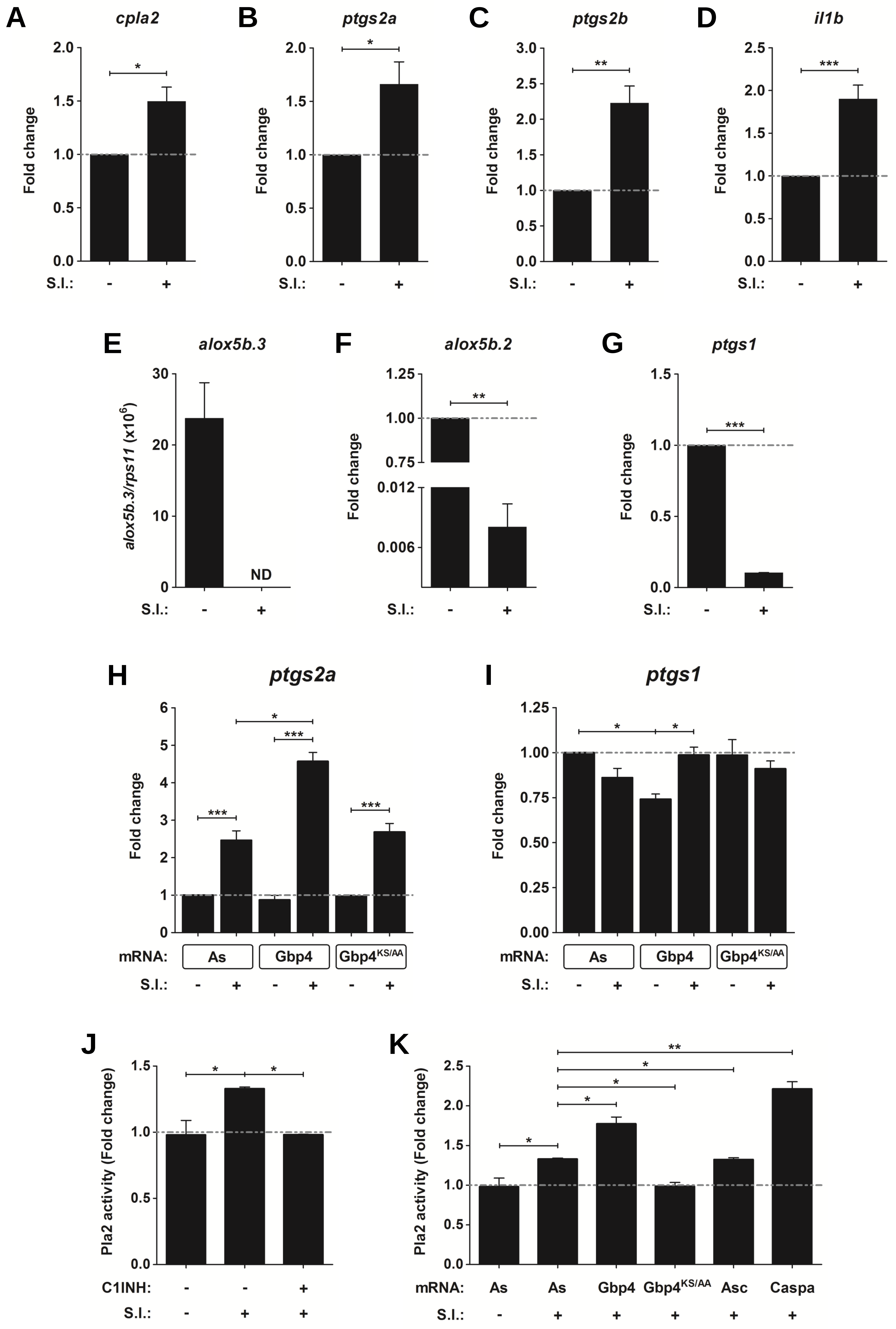

Supplementary Figure 11, related to Figures 8, 9. Disparate regulation of the expression of genes encoding the enzymes involved in eicosanoid biosynthesis by *S.* Typhimurium infection. (A, I) mRNA levels of the indicated genes were measured by RT-qPCR in FACS-sorted neutrophils from 3 dpf *mpx:eGFP* larvae (A-G) or whole wild type larvae (H, I), which were previously infected with ST or not at 2 dpf. Data were normalized with uninfected neutrophils. (J, K) Larvae were infected at 2 dpf with ST and the Pla2 activity in whole cell extracts determined using the EnzChek Phospholipase A2 Assay Kit. The sample size for each treatment is 950 in A-G, 25 in H and I, 30 in J and K. ND: non detected. *p<0.05; **p<0.01; ***p<0.001 according to Student t-test (A-G) or ANOVA followed by Tukey multiple range test (H-K).

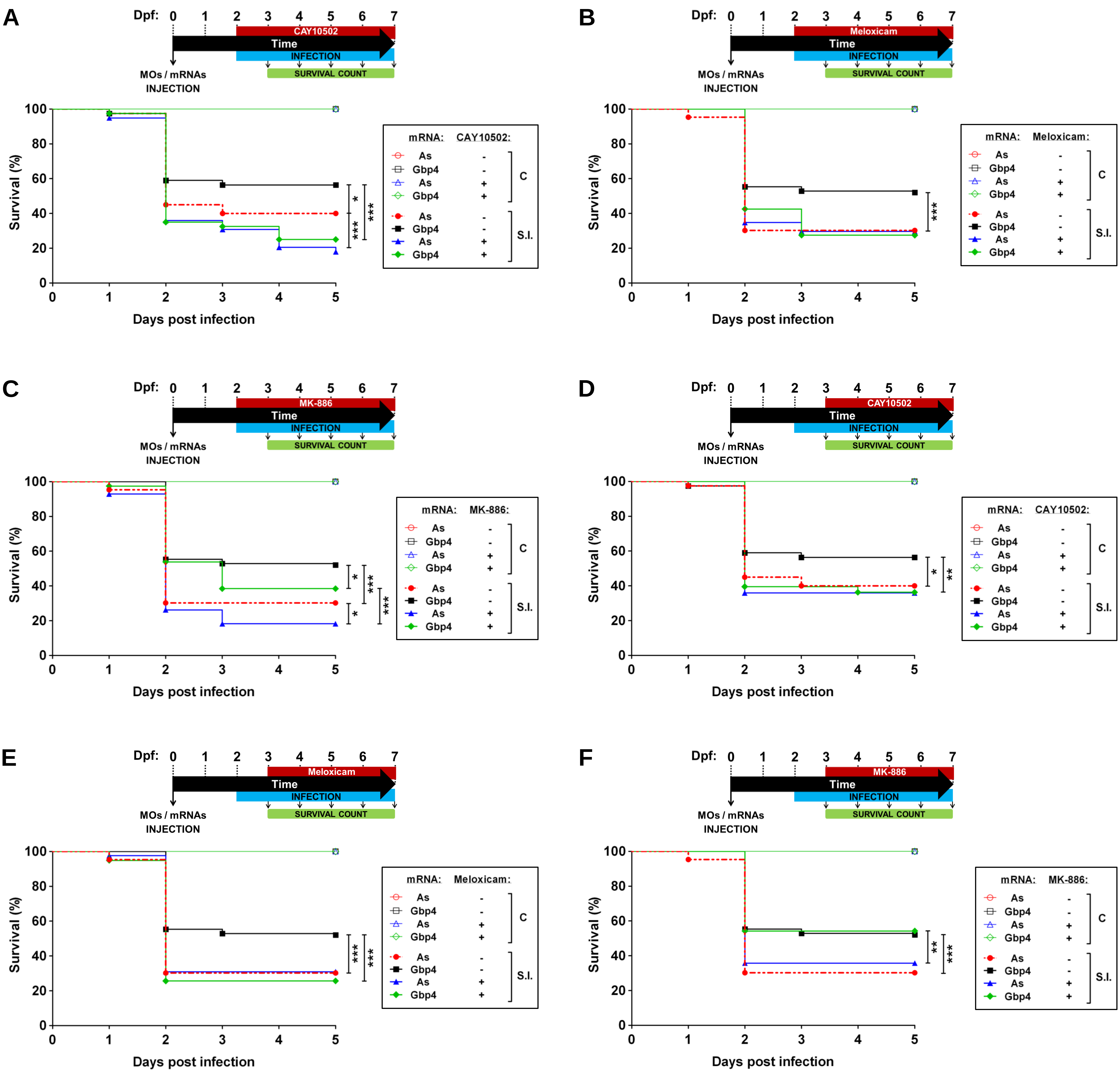

Supplementary Figure 12, related to Figures 8, 9. Impact of pharmacological inhibition of key enzymes involved in eicosanoid biosynthesis on larval resistance to *S.* Typhimurium infection. (A-F) Zebrafish one-cell embryos were injected with antisense (As) or Gbp4 mRNAs. Larvae were then treated by immersion with pharmacological inhibitors of cPla2 (CAY10502, 0.5 µM) (A, D), Ptgs2 (meloxicam, 10 µM) (B, E), Alox5 (MK-886, 1 µM) (C, F) or vehicle alone (DMSO) at 2 dpf (1 h before infection) (A-C) or 3 dpf (24 hpi) (D-F), infected at 2 dpf and survival determined as described in Figure 2A. The sample size for each treatment is 300 in A-F. S.I., ST infection. *p<0.05; **p<0.01; ***p<0.001 according to log rank test (A-F).

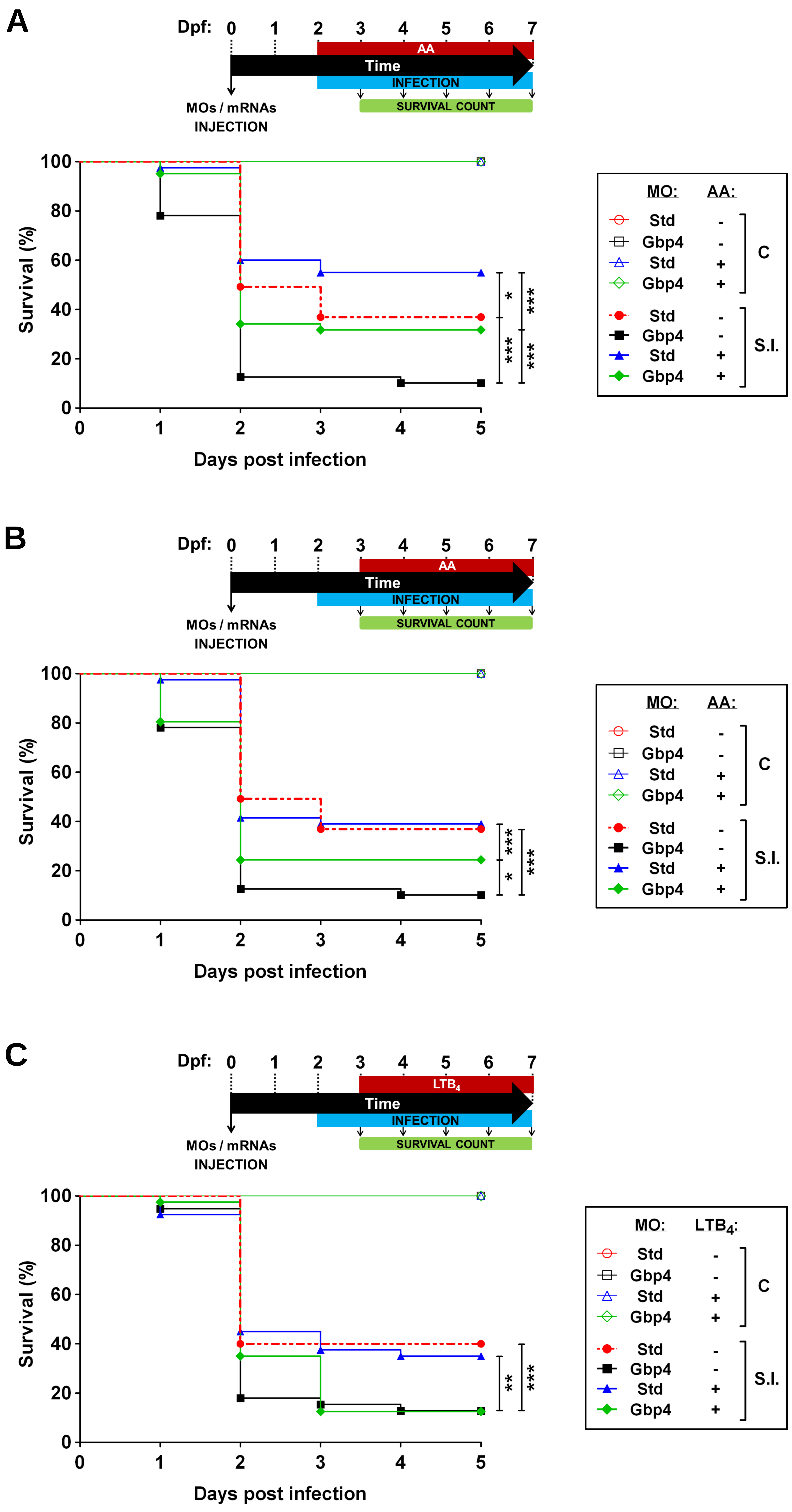

Supplementary Figure 13, related to Figures 8, 9. Exogenous addition of AA increases larval resistance to *S.* Typhimurium infection, while LTB4 does not affect the bacterial clearance phase. (A-C) Zebrafish one-cell embryos were injected with control (Std) or Gbp4 MOs. Larvae were then treated by immersion with 20 µM AA (A, B), 1 µM LTB4 (C) or vehicle alone (DMSO) at 2 dpf (1 h before infection) (A) or 3 dpf (24 hpi) (B, C), infected at 2 dpf and survival determined as described in Figure 2A. The sample size for each treatment is 300 in A-C. S.I., ST infection. *$p<0.05$; **$p<0.01$; ***$p<0.001$ according to log rank test (A-C).

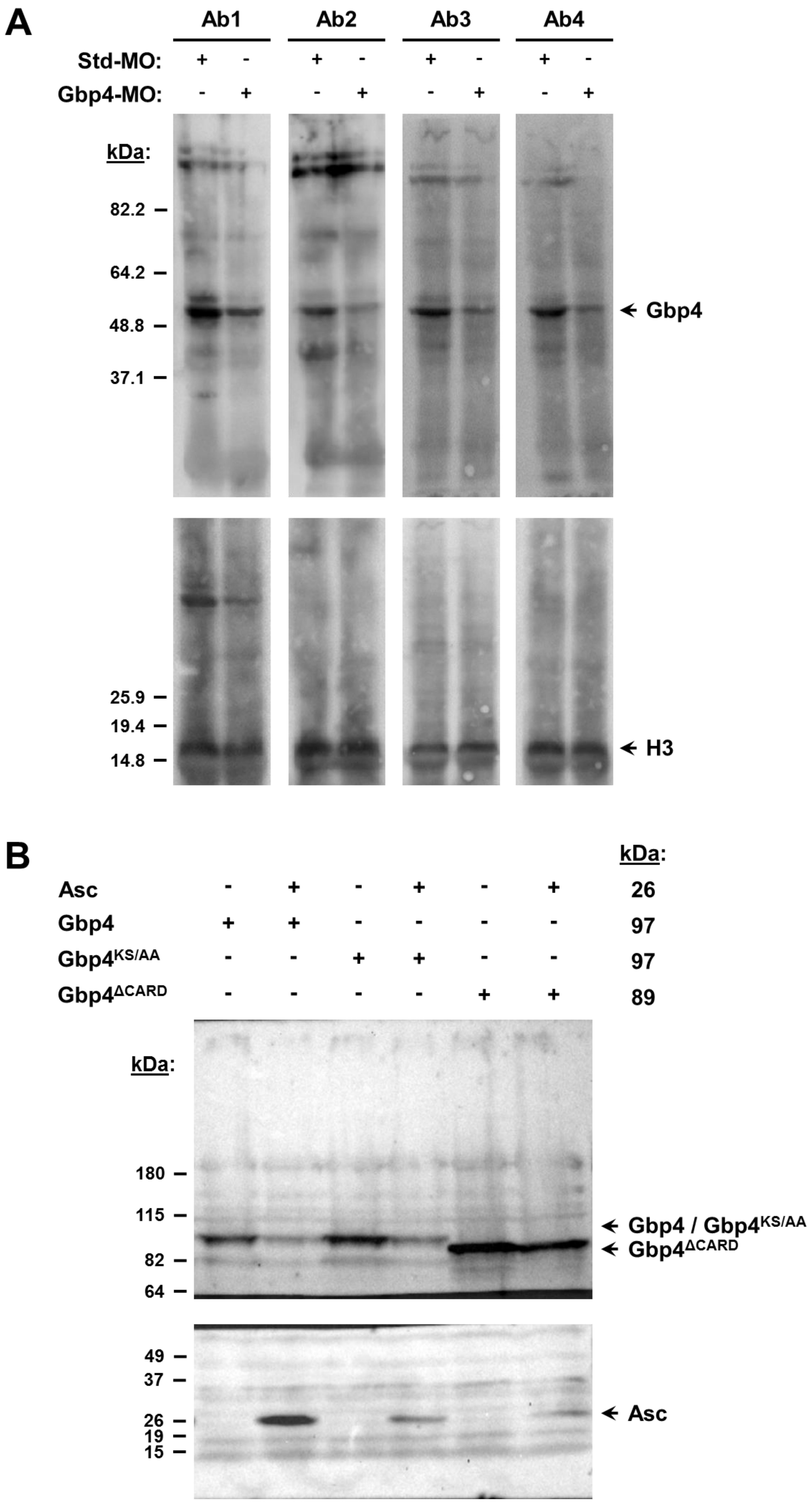

Supplementary Figure 14, related to Figs. 1 and 6. Full size images of western blots cropped for presentation in Figs. 1E (A) and 6E (B). The molecular weight ladders are also shown.

Supplementary Table 1. Lipidomic analysis in 3 dpf whole zebrafish larvae at 8 and 24 h post-injection (hpi) with PBS or *S*. Typhimurium (ST). Zebrafish one-cell embryos were injected with antisense (As), Gbp4 and Asc mRNAs, infected at 2 dpf and the levels of the indicated lipid mediators determined by LC-MS-MS. Lipid concentrations are shown in pg/1000 larvae. LT, leukotriene; LX, lipoxin; TX, thromboxane; PG, prostaglandin.

	LTB4	LXA4*	LXB4	PGD2	PGE2	PGF2α	TXB2	
**As / PBS**	0.9	1.8	34.3	0.5	1.1	3.1	0.2	**Neutrophil recruitment phase (8 hpi)**
**As / ST**	1.4	1.6	36.4	1.2	4.3	8.3	0.2	
**Gbp4 / ST**	1.4	9.8	37.1	1.0	6.1	8.9	0.2	
**As / PBS**	0.9	181.1	21.5	0.6	8.7	3.8	0.3	**Bacterial clearance phase (24 hpi)**
**As / ST**	1.1	241.3	31.4	1.3	17.8	11.1	0.3	
**Gbp4 / ST**	2.6	32.7	58.6	1.9	35.9	32.7	0.4	
**Asc / ST**	2.1	5.3	35.7	1.8	22.7	24.9	0.2	

*13,14-dihydro-15-oxo LXA4: LXA4 metabolite found in zebrafish[5].

Supplementary Table 2. Lipidomic analysis in 3 dpf whole zebrafish larvae at 24 h post-injection (hpi) with PBS or *S.* Typhimurium (ST). Zebrafish larvae were treated by immersion with vehicle alone (DMSO) or 100 µM of a specific inhibitor of caspase-1 (Ac-YVAD-CMK ,C1INH) at 2 dpf (1 h before infection), infected and the levels of the indicated lipid mediators determined by LC-MS-MS at 24 hpi. Lipid concentrations are shown in pg/1000 larvae. LT, leukotriene; LX, lipoxin; TX, thromboxane; PG, prostaglandin.

	LTB4	LXA4*	LXB4	PGD2	PGE2	PGF2α	TXB2	
**DMSO / PBS**	4.1	134.5	29.2	1.9	31.4	17.9	1.4	**Bacterial clearance phase (24 hpi)**
**DMSO / ST**	7.4	434.5	58.1	4.2	56.9	42.1	1.9	
**C1INH / ST**	4.7	157.6	38.2	0.3	11.0	11.0	1.1	

*13,14-dihydro-15-oxo LXA4: LXA4 metabolite found in zebrafish[5].

Supplementary Table 3. Morpholinos used in this study. The gene symbols followed the Zebrafish Nomenclature Guidelines (http://zfin.org/zf_info/nomen.html).

Gene	Ensembl ID	Target	Sequence (5'→3')	Concentration (mM)	Reference
gbp4	ENSDARG00000068857	e1/i1	GCTGTTTGTGTGTCTCTAACCTGTT	0.1	This work
asc (pycard)	ENSDARG00000040076	e2/i2	AGTGATTCGCTTACTCACCATCAGA	1.68	This work
		5'UTR/ATG	GCTGCTCCTTGAAAGATTCCGCCAT	0.6	This work
cxcr2	ENSDARG00000054975	5'UTR/ATG	ACTCTGTAGTAGCAGTTTCCATGTT	0.3	Deng *et al.*, 2013
il1b	ENSDARG00000098700	i2/e3	CCCACAAACTGCAAAATATCAGCTT	0.5	López-Muñoz et al., 2011
lta4h	ENSDART00000028171	5'UTR/ATG	AGCTAGGGTCTGAAACTGGAGTCAT	0.2	Tobin *et al.*, 2010

Supplementary Table 4. Primers used in this study. The gene symbols followed the Zebrafish Nomenclature Guidelines (http://zfin.org/zf_info/nomen.html). ENA, European Nucleotide Archive (http://www.ebi.ac.uk/ena/).

Gene	ENA ID	Name	Sequence (5'→3')	Use
asc (pycard)	NM_131495	F	ATTTTGAGGGCGATCAAGTG	RT-PCR
		R	GCATCCTCAAGGTCATCCAT	
rps11	NM_213377	F1	GGCGTCAACGTGTCAGAGTA	RT-qPCR
		R1	GCCTCTTCTCAAAACGGTTG	
gbp4	NM_001082945	F	ACTGGGAGATGTGGAAAAGGGCG	
		R	CCATAGCCTTGTTGTCGATCACCCC	
il1b	NM_212844	F5	GGCTGTGTGTTTGGGAATCT	
		R5	TGATAAACCAACCGGGACA	
gfp	EF591490	F1	ACGTAAACGGCCACAAGTTC	
		R1	AAGTCGTGCTGCTTCATGTG	
ptgs2b	NM_001025504	F2	CCCCAGAGTACTGGAAACCA	
		R2	ACATGGCCCGTTGACATTAT	
ptgs2a	NM_153657	F1	TGGATCTTTCCTGGTGAAGG	
		R1	GAAGCTCAGGGGTAGTGCAG	
ptgs1	AY028584	F	TTTTGCTGCTGAGTGTGTCC	
		R	CGAACACAGATCCCTTGGTT	
cpla2	NM_131295	F1	CTGTTCATGCAGACACGCAG	
		R1	GGTGGGAACCTCTCTTGGTG	
alox5b.2	NM_001045331	F1	GGACAACGAGCTGTTTTTAGGC	
		R1	CATTTCGGCTCCTGATGGTCT	
alox5b.3	NM_001020578	F1	TCAGAGGCGTCATCAAGAGC	
		R1	CGTCAATGGAAATCACCGTTCC	

Supplementary References

1. Martinon, F., Burns, K. & Tschopp, J. The inflammasome: a molecular platform triggering activation of inflammatory caspases and processing of proIL-beta. *Mol Cell* **10**, 417-426 (2002).
2. Matsushita, K. *et al.* A splice variant of ASC regulates IL-1beta release and aggregates differently from intact ASC. *Mediators Inflamm* **2009**, 287387 (2009).
3. Robert, X. & Gouet, P. Deciphering key features in protein structures with the new ENDscript server. *Nucleic Acids Res* **42**, W320-324 (2014).
4. Pettersen, E.F. *et al.* UCSF Chimera--a visualization system for exploratory research and analysis. *J Comput Chem* **25**, 1605-1612 (2004).
5. Tobin, D.M. *et al.* Host genotype-specific therapies can optimize the inflammatory response to mycobacterial infections. *Cell* **148**, 434-446 (2012).
